# Novel synthesized triazole derivatives as effective corrosion inhibitors for carbon steel in 1M HCl solution: experimental and computational studies

**DOI:** 10.1038/s41598-023-49468-5

**Published:** 2023-12-13

**Authors:** Kamelia Belal, A. H. El-Askalany, Eslam A. Ghaith, Ahmed Fathi Salem Molouk

**Affiliations:** 1 Department of Chemistry, Faculty of Science, Mansoura University, Mansoura, 35516 Egypt; 2 Faculty of Science, New Mansoura University, New Mansoura City, Egypt

**Keywords:** Chemical engineering, Electrochemistry, Physical chemistry

## Abstract

This article outlines the synthesis of two derivatives of 4-amino-5-hydrazineyl-4*H*-1,2,4-triazole-3-thiol for the prevention of carbon steel corrosion in 1M HCl solution. These derivatives are (*Z*)-3-(1-(2-(4-amino-5-mercapto-4*H*-1,2,4-triazol-3-yl)hydrazono)ethyl)-2*H*-chromen-2-one (**TZ1**) and 5-(2-(9*H*-fluoren-9-ylidene)hydrazineyl)-4-amino-4*H*-1,2,4-triazole-3-thiol (**TZ2**). Weight loss, electrochemical experiments, surface examinations, and theoretical computation are used to evaluate the effectiveness of the two compounds to be used as corrosion inhibitors. Weight loss and electrochemical studies demonstrate that these derivatives reduce the corrosion rate of carbon steel. To examine the morphology and constitution of the carbon steel surface submerged in HCl solution as well as after adding inhibitors, surface examination tests are performed. Analysis of the test solution via UV–visible spectroscopy is employed to check the possibility of complex formation between inhibitor molecules and Fe^2+^ ions released during the corrosion process. In order to explore their biological activity, the antibacterial activity was investigated against (*E. coli* and *Bacillus subtilis*). Finally, theoretical confirmation of the experimental findings is provided by quantum chemical (DFT) and Monte Carlo (MC) simulation studies. More adsorption sites are present in the derivatives of 4-amino-5-hydrazineyl-4*H*-1,2,4-triazole-3-thiol, which offer a novel perspective for developing new classes of corrosion inhibitors with substantial protective efficacy, especially at high temperatures.

## Introduction

Carbon steel plays a crucial role in water supply systems, machine–equipment, metal smelting, building constructions, petroleum, and electric industries due to its exceptional ductility, weldability qualities, thermal and electrical conductivity. However, carbon steel was vulnerable to corrosion in acidic environments like descaling, pickling, and acidizing oil wells, which led to several issues such as production halts, environmental contamination, and the consumption of resources^1^. Microbes are also grown concurrently with industrial development^2^.

One of the most popular and efficient ways to avoid metal corrosion is by adding corrosion inhibitors to acid solutions because of the technical advancement, affordability, and ease of usage. Organic corrosion inhibitors can be adsorbed on metal surfaces and act as a protective barrier between metals and acid solutions, therefore reducing the corrosion rate^3^. However, the toxicity of anticorrosion compounds is still a problem. This motivates scientists to develop corrosion inhibitors that have superior inhibition efficiency and no toxic units^4^.

It has been reported that compounds containing heteroatoms, such as S, N, P, and O, and/or conjugated systems, are efficient at mitigating the corrosion of metals^5–7^. Nitrogenous heterocyclic scaffolds have excellent corrosion inhibition potential in different circumstances, such as sour, scaling environments, and acid pickling due to their capability to coordinate and bond with metallic substrates^8,9^.

Researchers have tested a variety of triazoles as they are effective in mitigating metal corrosion^10–14^. Additionally, triazoles have been regarded as cost-effective, easily synthesized, and environmentally beneficial substances. Numerous triazole compounds have a wide range of pharmacological functions, including antioxidant, anti-bacterial, anti-depressant, anti-tubercular, anti-inflammatory, anti-neoplastic, and anticonvulsant activities^15–17^. This proves that triazole compounds are non-toxic and environmentally friendly^18–20^. As a result, researchers have worked hard to look at several novel triazole derivatives to increase the efficiency of its inhibition. A common method for improving a given heterocycle’s ability to suppress corrosion is to change its structure by adding new moieties or functional groups resulting in a rise in aromaticity, electron density, and active sites.

Accordingly, this work discusses the design and synthesis of two novel triazole derivatives, namely, (*Z*)-3-(1-(2-(4-amino-5-mercapto-4*H*-1,2,4-triazol-3-yl)hydrazono)ethyl)-2*H*-chromen-2-one(**TZ1**) and 5-(2-(9*H*-fluoren-9-ylidene)hydrazinyl)-4-amino-4*H*-1,2,4-triazole-3-thiol(**TZ2**). These derivatives were selected because they are easy to prepare with lower cost, and contain several active centers. Moreover, coumarin compounds are being employed extensively as corrosion inhibitors and exhibit high biological and pharmaceutical effects^21,22^ which implies that these compounds are environmentally safe and promising inhibitors. Therefore, the originality of this study relies on the incorporation between 4-amino-5-hydrazineyl-4*H*-1,2,4-triazole-3-thiol and Coumarin or Fluorenone for inhibitors **TZ1** and **TZ2**, respectively in an attempt to increase the active sites, aromaticity, and electron density to design novel inhibitors that are not discussed before as protecting material for CS corrosion in 1.0 M HCl. Besides, this work targeted the use of low-concentration content of the newly synthesized triazole compounds with inhibition efficiency comparable to that found for other triazole derivatives in the literature in order to be more cost-effective. Therefore, the performance of the designed inhibitors was tested towards carbon steel corrosion in 1.0 M HCl by utilizing data collected from weight loss (WL), Potentiodynamic polarization (PP), as well as electrochemical impedance spectroscopy (EIS). The morphology of the CS surface was obtained via atomic force microscopy (AFM) and X-ray Photoelectron spectroscopy (XPS) measurements, and analysis of the test solution via UV–visible spectroscopy. In addition, **TZ1** and **TZ2** have been evaluated "in vitro" for their antibacterial activity using bacterial strains: *E. coli* and *Bacillus subtilis*. The experimental findings were further supported via theoretical quantum calculations (DFT) and Monte Carlo (MC) simulations.

## Experimental part

### Materials

The composition of CS samples in (weight%) is: carbon 0.2%, manganese 0.5%, sulphur 0.05%, Silicon 0.25%, and iron 99%.

### Synthesis of inhibitors

**Figure 1:**
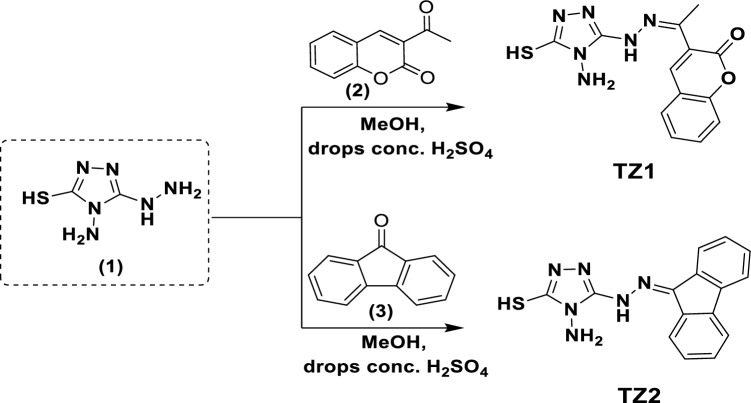
Synthetic routes of **TZ1** and **TZ2** molecules.

**TZ1** and **TZ2** molecules were synthesized in accordance with Fig. 1.

#### General procedure for synthesis of inhibitors

A mixture of 4-amino-5-hydrazineyl-4*H*-1,2,4-triazole-3-thiol (**1**) (0.146 g, 1 mmol) and 3-acetyl-coumarine (**2**) (0.188 g, 1 mmol) or 9*H*-fluoren-9-one (**3**) in methanol (20 ml) containing 3–4 drops of conc. H_2_SO_4_ was refluxed for 10 min. The formed precipitate was obtained on hot, then filtrated and washed with hot methanol (20 ml) to remove unreacted starting materials. Finally, the formed precipitates of compounds **TZ1** and **TZ2** were dried in the oven at 80 °C. On the other hand, ^1^H, D_2_O, and ^13^C NMR spectra of the synthesized compounds were depicted in Figs. S1–S6 in the supplementary file.

##### (*Z*)-3-(1-(2-(4-amino-5-mercapto-4*H*-1,2,4-triazol-3-yl)hydrazono)ethyl)-2*H*-chromen-2-one (TZ1)

Yield, 94%; orange sheets; m.p 288–290 °C; [DMF: EtOH (2:1)]; R_f_ = 0.35 [pet ether: ethyl acetate (1:1)]; IR (KBr) νmax, cm^−1^: 3402, 3309 (NH_2_), 3126 (NH), 1713 (C = O, lactonic), 1639, 1605 (C = N), 1514 (CH_Arom_); ^1^H NMR (DMSO-*d*_*6*_, 500 M *Hz*): δ (ppm) 2.24 (s, 3H, CH_3_), 3.45 (br, 2H, NH_2_, exchangeable D_2_O), 7.37 (t, 1H, *J* = 5 *Hz*), 7.43 (d, 1H, *J* = 8 *Hz*), 7.63 (t, 1H, *J* = 7.2 Hz), 7.84 (d, 1H, *J* = 6.5 *Hz*), 8.18 (s, 1H, CH_olefinic_), 9.26 (s, 1H, NH, exchangeable D_2_O), 13.09 (s, 1H, SH, exchangeable D_2_O); ^13^C NMR (DMSO-*d*_*6*_, 125 M*Hz*): *δ* (ppm) = *δ* 164.2, 159.2, 153.3, 149.7, 148.1, 141.0, 132.2, 129.1, 126.3, 124.7, 118.8, 115.9, 15.3.

##### 5-(2-(9*H*-fluoren-9-ylidene)hydrazineyl)-4-amino-4*H*-1,2,4-triazole-3-thiol (TZ2)

Yield, 86%; red powder; m.p 264–266 °C; [DMF: EtOH (2:1)]; R_f_ = 0.46 [pet ether: ethyl acetate (1:1)]; IR (KBr) νmax, cm^−1^: 3417, 3306 (NH_2_), 3139 (NH), 1638, 1610 (C = N), 1498 (CH_Arom_); ^1^H NMR (DMSO-*d*_*6*_, 500 M *Hz*): δ (ppm) 5.64 (s, 2H, NH_2_, exchangeable D_2_O), 7.35 (t, *J* = 7.2 Hz, 1H), 7.42–7.48 (m, 2H), 7.55 (t, *J* = 7.7 Hz, 1H), 7.74 (d, *J* = 8 Hz, 1H), 7.85 (d, 1H, *J* = 7.5 Hz), 7.94 (d, 1H, *J* = 7 Hz), 8.11 (d, 1H, *J* = 8 Hz), 10.13 (s, 1H, NH, exchangeable D_2_O), 13.23 (s, 1H, SH, exchangeable D_2_O); ^13^C NMR (DMSO-*d*_*6*_, 125 MHz): *δ* (ppm) = *δ* 164.8, 149.9, 148.3, 141.0, 138.9, 136.3, 131.1, 129.9, 129.3, 128.2, 128.1, 126.7, 121.3, 120.9, 120.3.

### Aqueous solutions

The corrosive medium, 1 M HCl, was prepared by diluting 37% HCl analytical grade (Acros Organics Brand supplied by Cornell Lab, Egypt) with double-distilled water. This concentration was standardized using a standard solution of sodium carbonate. Stock solutions (10^−3^ M) were prepared for each of **TZ1** and **TZ2** by dissolving the appropriate amount of solid in 10 ml dimethyl-sulfoxide (DMSO) then the volume was completed to 100 ml with absolute ethanol. The resulting stock solutions were diluted using double-distilled water to the needed concentration range (1–9 × 10^−5^ M). To negate the influence of solvents on the inhibition, the percentage of solvents in which the inhibitor dissolved was kept constant throughout the prepared solutions in both the presence and absence of the inhibitors.

### WL method

Six CS samples with dimensions 2.2 × 1.6 × 0.2 cm (L × W × H) were pretreated and polished by utilizing different grades of emery paper (400, 1000, 1200, and 2000), cleaned with double-distilled water, dehydrated by filter papers, and weighed. Then, samples were dipped into 1M HCl solution using a glass hook without the use of inhibitors (**TZ1**, **TZ2)** and after adding different concentrations for 6 h at various temperatures (25–45 °C). The samples were withdrawn, rinsed, dried, and weighed again every hour. The inhibition efficiency (%IE) as well as the surface coverage (θ) of the studied inhibitor molecules were computed using Eq. (1)^23^.

1 $$\% {\text{IE}} =\uptheta \times {1}00 = \left( {1 - \frac{{\text{W}}}{{{\text{ W}}^{{\circ }} }}} \right) \times 100,$$

where (W°) and (W) are the average weight loss without as well as with the inhibitor, respectively.

### Electrochemical techniques

A Potentiostat/Galvanostat/ZRA analyzer (Gamry 5000E, USA) was utilized to conduct all the electrochemical tests. Three electrodes were employed in the glass cell: a working electrode (CS sample with an exposed surface area of 0.8 cm^2^), an auxiliary electrode (platinum sheet), and a reference electrode (Ag/AgCl electrode). The samples were pre-treated like the WL method. At 25 °C, these electrodes were immersed in 1M HCl solution both before and after different concentrations of the inhibitors were added. PP curves were acquired using a voltage range of ± 500 mV at E_ocp_ with a scanning rate of 0.5 mV/s. The extrapolation of the cathodic and anodic (β_c_ and β_a_) Tafel slopes of the curves yielded the corrosion current (i_corr_) value. To obtain %IE and θ, Eq. (2) was applied. To carry out the electrochemical impedance spectroscopy (EIS) test, a frequency range of 0.1–100,000 Hz and an amplitude of 10 mV were applied. From Eq. (3), %IE and θ were obtained^24^.

2 $$\% {\text{IE}} =\uptheta \times {1}00 = \left( {1 - \frac{{{\text{i}}_{{{\text{corr}}}} }}{{{\text{i}}^\circ_{{{\text{corr}}}} }}} \right) \times {1}00,$$

where (i°_corr_) and (i_corr_) represent the corrosion current densities without as well as after utilizing the inhibitor, respectively.

3 $$\% {\text{IE}} =\uptheta \times {1}00 = \left( {1 - \frac{{{\text{R}}^\circ_{{{\text{ct}}}} }}{{{\text{R}}_{{{\text{ct}}}} }}} \right) \times {1}00,$$

where (${{\text{R}}^\circ }_{{\text{ct}}}$) and (${{\text{R}}}_{{\text{ct}}}$) represent the charge transfer resistance without as well as after utilizing the inhibitor, respectively.

### Surface examinations

#### AFM analysis

The CS sheets were polished until the surface appeared like a mirror, rinsed, dried, and then dipped in HCl solution without as well as with the existence of 9 × 10^−5^ M of **TZ1** and **TZ2** at 25 °C for 24 h. Following removal from the solution, the CS samples were rinsed, dried, and subjected to AFM examination (Nanosurf FlexAFM 3, Gräubernstrasse 12, 4410 Liestal, Switzerland). This approach was applied at the Nanotechnology laboratory, Faculty of Engineering, Mansoura University to investigate how the tested inhibitors affected the morphology of the metal surfaces in 1M HCl.

#### XPS analysis

The CS samples were pre-treated first as AFM analysis. XPS analysis was performed to determine the composition of the adsorbed layers on the surface of CS by using (AXIX Ultra DLD, Kratos, UK).

### Solution analysis (UV–visible spectroscopy)

This technique was carried out to examine the complexation between Fe^2+^ ions released during the corrosion process and the inhibitors. (T80+ UV/vis spectrometer, UK) was used to record the spectra.

### Antibacterial activity test

The agar well diffusion method was used to evaluate the antibacterial activity of **TZ1** and **TZ2**^25,26^. The tested organisms are gram-negative bacteria (*Escherichia coli*) and gram-positive bacteria (*coli*). The agar plate surface is inoculated by spreading a volume of the microbial inoculum over the entire agar surface. Then, a hole with a diameter of 9 mm is punched aseptically with a sterile cork borer or a tip, and a volume (50 µL) of the antibacterial agent at the desired concentration is introduced into the well. Then, agar plates are incubated under suitable conditions depending on the test microorganism. After the incubation, inhibition zones for the antibacterial agents were measured in diameter.

### DFT and MC simulation studies

The DMol^3^ module in Materials Studio 2017 and the GGA technique with a DNP basis set as well as BOP functional incorporates COSMO controls were both used to perform quantum chemical calculations in the aqueous phase^27,28^. While using the Adsorption Locator module, MC simulation was carried out to identify the adsorption configurations of the two tested inhibitors on the interface of Fe (110)^29^. All computations were performed utilizing the force field COMPASS (Condensed-phase Optimized Molecular Potential for Atomistic Simulation Study)^30^.

## Results and discussion

### WL Measurements

#### Impact of concentrations and temperature

**Figure 2:**
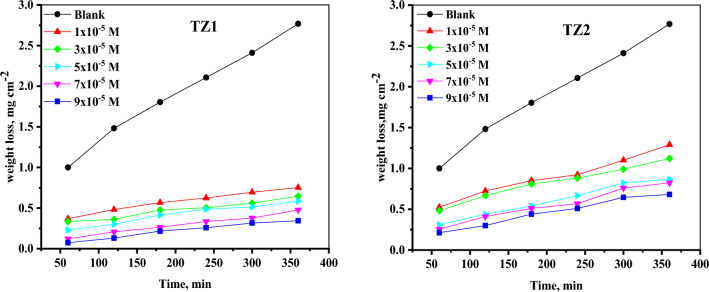
WL-Time curves for CS in 1 M HCl without and with the existence of different concentrations of **TZ1** and **TZ2** at 25 °C.

Using the WL approach at various temperatures, the corrosion rate of CS in 1M HCl solution as well as inhibited 1M HCl with varying concentrations of the inhibitors was studied. WL at 25°C using various concentrations of inhibitor molecules (**TZ1** and **TZ2**) varies over time as seen in Fig. 2, whereas Table 1 displays how temperature affects %IE and CR. As the concentration of inhibitor in the test solution increased, it was discovered that the CR clearly decreased, causing the %IE to rise. These findings imply that **TZ1** and **TZ2** are influential inhibitors for CS corrosion in HCl solution. Additionally, as the inhibitor concentrations were increased, the surface coverage on the CS surface would rise^31,32^. A close examination of the data summarized in Table 1 revealed that, for the same inhibitor concentration, the inhibition efficiencies are in the following order: **TZ1** > **TZ2, c**onsequently **TZ1** is the most efficient inhibitor. The influence of temperature on the rate of CS corrosion in 1M HCl as well as with the inclusion of various concentrations of **TZ1** and **TZ2** was examined between 25 and 45 °C with 5-degree increments. Raising the temperature improves the inhibitory performance of **TZ1** and **TZ2**, as seen in Table 1, suggesting that the kind of adsorption could be chemisorption^33^.

**Table 1:** Values of CR and % IE of **TZ1** and **TZ2** for CS corrosion in 1M HCl estimated from WL measurements using different concentrations at 25–45 °C.

Inhibitor	Conc (M)	25 °C		30 °C		35 °C		40 °C		45 °C	
		CR	%IE	CR	%IE	CR	%IE	CR	%IE	CR	%IE
Blank	1M HCl	0.0080	–	0.0115	–	0.0170	–	0.0277	–	0.0430	–
**TZ1**	1 × 10^−5^	0.0023	71.2	0.0031	72.9	0.0041	75.8	0.0062	77.6	0.0089	79.3
	3 × 10^−5^	0.0019	76.7	0.0026	77.2	0.0037	78.3	0.0048	82.7	0.0062	85.7
	5 × 10^−5^	0.0017	78.7	0.0023	80.3	0.0029	82.8	0.0043	84.5	0.0055	87.3
	7 × 10^−5^	0.0013	84.4	0.0016	85.7	0.0023	86.4	0.0032	88.6	0.0040	90.8
	9 × 10^−5^	0.0011	86.9	0.0015	87.1	0.0020	88.0	0.0025	91.0	0.0027	93.7
**TZ2**	1 × 10^−5^	0.0037	54.4	0.0050	56.4	0.0070	58.5	0.0111	60.0	0.0148	65.6
	3 × 10^−5^	0.0033	58.9	0.0045	60.9	0.0065	61.4	0.0093	66.3	0.0124	71.1
	5 × 10^−5^	0.0027	65.9	0.0037	67.9	0.0048	71.5	0.0066	76.3	0.0093	78.5
	7 × 10^−5^	0.0025	68.5	0.0034	70.8	0.0042	75.1	0.0057	79.5	0.0071	83.5
	9 × 10^−5^	0.0022	73.2	0.0029	74.9	0.0039	77.0	0.0052	81.2	0.0067	84.5

#### Thermodynamic activation parameters

**Figure 3:**
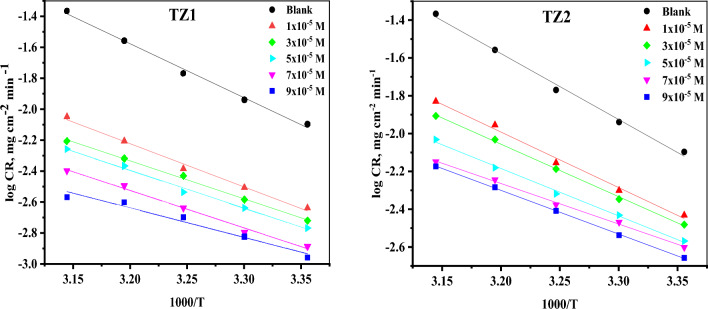
Arrhenius plots for the corrosion of CS in 1M HCl without as well as after utilizing different concentrations of **TZ1** and **TZ2**.

**Figure 4:**
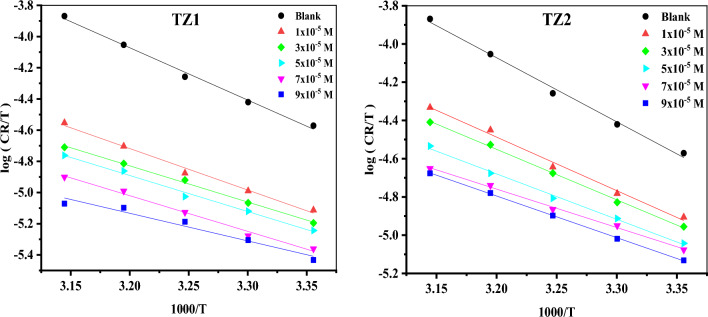
Transition state plots for the corrosion of CS in 1M HCl without as well as after utilizing different concentrations of **TZ1** and **TZ2**.

According to the Arrhenius equation Eq. (4), the activation parameters for the corrosion process of CS were computed^34^and listed in Table 2.

4 $$\mathrm{log CR}=\mathrm{log A}-\frac{{{E}^{*}}_{{\text{a}}}}{2.303{\text{RT}} },$$

where (E_a_^*^) represents the apparent activation energy, A denotes the frequency factor, R is the universal gas constant, T represents the absolute temperature and CR denotes the corrosion rate computed via WL measurements. Figure 3 displays Arrhenius plots for **TZ1** and **TZ2**. Straight lines with a slope of (− E_a_*/2.303R) and an intercept (log A) were established. The E_a_* values in Table 2 decreased as the inhibitor concentration increased, demonstrating chemisorption adsorption^35^. The transition state equation was employed to estimate the enthalpy as well as the entropy of activation (ΔH*and ΔS*) Eq. (5)^36^.

5 $${\text{log}}\left( {{\text{CR}}/{\text{T}}} \right) = {\text{log}}\left( {{\text{R}}/{\text{Nh}}} \right) + \left( {\Delta {\text{S}}*/{2}.{3}0{\text{3R}}} \right) + \left( { - \Delta {\text{H}}*/{2}.{3}0{\text{3RT}}} \right),$$

where N = Avogadro’s number, h refers to Planck's constant, ΔH^*^ and ΔS^*^ are the enthalpy and the entropy of activation, respectively. The transition state plots for **TZ1** and **TZ2** are shown in Fig. 4. Straight lines were gained with (slopes = − ΔH*/2.303R) and (intercepts = (log(R/Nh) + (ΔS^*^/2.303R)), which used for the computation of ∆H* and ∆S* as listed in Table 2. The positive values of ∆H* demonstrate that CS dissolves endothermically^37^. The activated complex more frequently existed in the associated form (**TZ1** and **TZ2** adsorbed on CS surface) than in the dissociated form (**TZ1** and **TZ2** in solution), as indicated by the negative values of ∆S*, suggesting that the disorder is reduced during the corrosion of CS^38^.

**Table 2:** The activation parameters for the corrosion of CS in 1M HCl without as well as after utilizing different concentrations of **TZ1** and **TZ2**.

Inhibitor	Conc (M)	Ea* (KJ mol^−1^)	ΔH* (KJ mol^−1^)	− ΔS* (J mol^−1^ K^−1^)
Blank	1 M HCl	66.66	64.10	70.44
**TZ1**	1 × 10^−5^	53.50	50.94	124.90
	3 × 10^−5^	46.98	44.43	147.87
	5 × 10^−5^	46.85	44.29	149.49
	7 × 10^−5^	46.34	43.78	153.59
	9 × 10^−5^	36.48	33.92	187.30
**TZ2**	1 × 10^−5^	56.23	53.67	111.75
	3 × 10^−5^	53.17	50.61	122.76
	5 × 10^−5^	48.07	45.51	141.55
	7 × 10^−5^	41.06	38.50	165.50
	9 × 10^−5^	44.31	41.75	155.78

#### Adsorption isotherms

**Figure 5:**
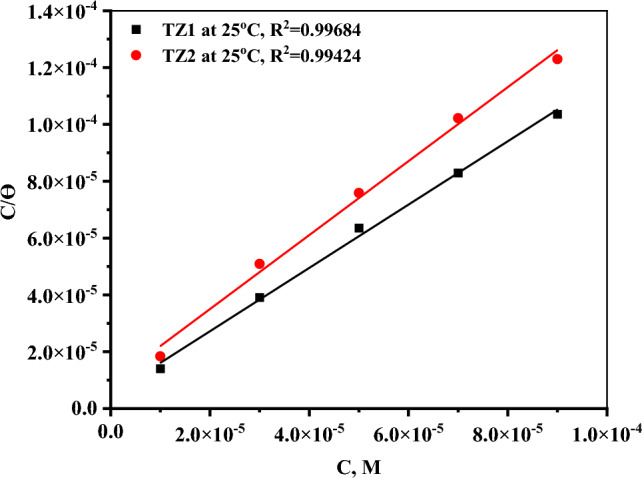
Langmuir adsorption isotherm of **TZ1** and **TZ2** for the corrosion of CS utilizing 1M HCl at 25 °C.

**Figure 6:**
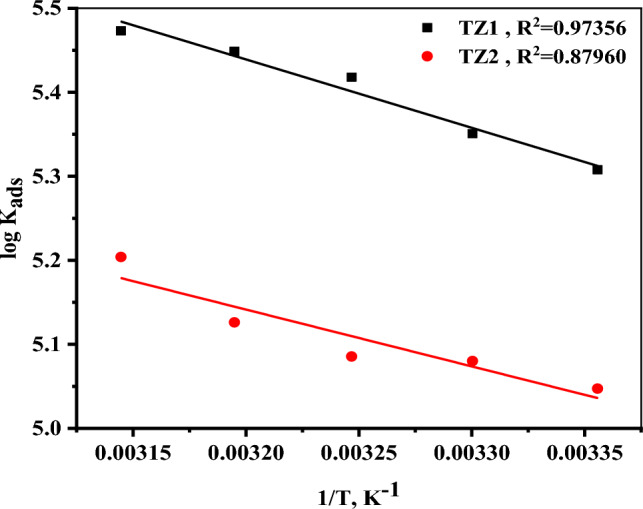
log K_ads_ versus (1/T) curves of **TZ1** and **TZ2**.

There are numerous adsorption isotherms that regulate how the two examined inhibitors interact with the CS surface. In order to know which adsorption model is in good agreement with the collected data, the relations of the most popular ones including Langmuir, Frumkin, Freundlich, El-Awady, Temkin, and Flory–Huggins isotherms were plotted, and then calculating the regression coefficient, slope, and intercept (see supplementary file S7 and Table S1)^39^. The values of the regression coefficient (R^2^) of Langmuir isotherm have least deviated from unity (R^2^) > 0.99. As a result, the adsorption of **TZ1** and **TZ2** molecules follows the Langmuir adsorption isotherm which was expressed by Eq. (6) that gave the best linear plots based on the fitted experimental data.

6 $${\text{C}}/\uptheta = 1/{\text{K}}_{{{\text{ads}}}} + {\text{C}},$$

where C and Ө are the inhibitor concentration (M) and the degree of surface coverage, respectively and K_ads_ is the adsorption equilibrium constant (M^-1^) that was calculated from the intercepts of the linear plots of C/θ against C, as shown in Fig. 5. The slopes and correlation coefficients (R^2^) for these plots were found to be close to unity. The acquired K_ads_ values (Table 3) for **TZ1** and **TZ2** gradually increase with increasing inhibitor concentration, implying excellent inhibition efficacy^40^. The values of standard free energy of adsorption (ΔG°_ads_) were computed based on K_ads_ values according to Eq. (7).

7 $$\Delta {\text{G}}^{{\circ }}_{{{\text{ads}}}} = - {2}.{3}0{3}\,{\text{RT}}\,{\text{log}}\left( {{\text{K}}_{{{\text{ads}}}} \times {55}.{5}} \right),$$

where 55.5 denotes the concentration of water in the solution (M), R is the universal gas constant (8.314 J K^−1^ mol^−1^), as well as T refers to the absolute temperature(kelvin). To comprehend the adsorption type of inhibitors, the value of ΔG°_ads_ was utilized. The values of ΔG°_ads_ are higher negative, as can be observed in Table 3, indicating that the adsorption of inhibitor molecules on the surface of CS is spontaneous^41^. According to the published papers, the adsorption of an inhibitor is referred to as physisorption if the ΔG°_ads_ values are close to − 20 kJ mol^−1^ or less negative, and chemisorption if they are close to − 40 kJ mol^−1^ or more negative^42,43^. The ΔG°_ads_ values for **TZ1** and **TZ2** range from − 38.76 to − 43.94 kJ mol^−1^, demonstrating that the inhibitors' adsorption mechanism on CS surface in 1M HCl solution appears to be mixed one (chemisorption and physisorption) but mainly chemisorption^44^. Equation (8) (Van’t Hoff equation) may be utilized for the computation of the heat of adsorption (ΔH°_ads_) by plotting log K_ads_ versus 1/T as displayed in Fig. 6^45^.

8 $${\text{log}}\,{\text{K}}_{{{\text{ads}}}} = \frac{{ - {\Delta H}^\circ { }_{{{\text{ads}}}} }}{{2.303{\text{RT}}}} + {\text{constant}},$$

**Table 3:** Adsorption isotherm parameters for **TZ1** and **TZ2** on the surface of CS utilizing 1M HCl at various temperatures.

Inhibitor	Temp (°C)	K_ads_ × 10^4^ (M^−1^)	− ΔG°_ads_ (KJ mol^−1^)	ΔH°_ads_ (KJ mol^−1^)	ΔS°_ads_ (J mol^−1^ K^−1^)
**TZ1**	25	20.3	40.23	15.59	187.32
	30	22.4	41.15		187.26
	35	26.2	42.23		187.73
	40	28.1	43.10		187.51
	45	29.7	43.94		187.20
**TZ2**	25	11.2	38.76	12.96	173.56
	30	12.0	39.58		173.40
	35	12.2	40.28		172.86
	40	13.4	41.17		172.94
	45	16.0	42.30		173.77

The standard adsorption entropy (ΔS°_ads_) can be assessed from Eq. (9)^45^.

9 $${\Delta G}^\circ { }_{{{\text{ads}}}} = {\Delta H}^\circ_{{{\text{ads}}}} - {\text{T}}\Delta {\text{S}}^\circ_{{{\text{ads}}}} ,$$

As shown in Table 3, the positive ΔH° _ads_ values imply that **TZ1** and **TZ2** adhered to the CS surface through an endothermic process. Endothermic adsorption is commonly believed to be caused by chemisorption as reported in previous works^46^. The positive sign of ΔS°_ads_ is due to the substitution process, which is caused by an increase in entropy at the CS/solution interface during the adsorption process because more water molecules are being desorbed from the metal surface by the inhibitor molecules existing in the solution^41^.

### Electrochemical measurements

#### PP Measurements

**Figure 7:**
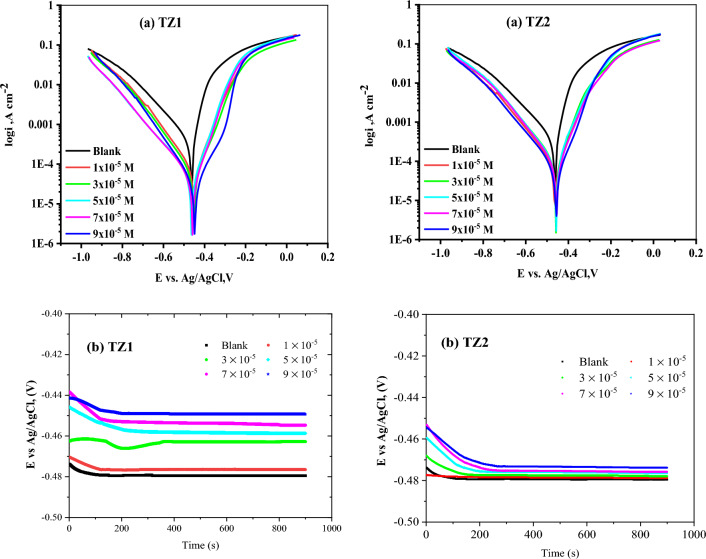
PP (**a**) and OCP (**b**) curves for the corrosion of CS in 1M HCl without as well as after utilizing different concentrations of **TZ1** and **TZ2** at 25°C.

Figure 7 displays PP (a) and open circuit potential (OCP) (b) curves for CS in 1M HCl without as well as after the inclusion of different concentrations of **TZ1** and **TZ2** at 25 °C, as well as the evaluated parameters were summarized in Table 4. The extrapolation of the anodic and cathodic curves yields the current density (i_corr_) and corrosion potentials (E_corr_) at the connecting point^47^. As depicted in Fig. 7a, the gradual addition of **TZ1** and **TZ2** led to a decrease in the current density of the anodic and cathodic reactions for CS in comparison to the blank solution and an increase in %IE. The fact that neither the cathodic Tafel slopes (β_c_) nor the anodic Tafel slopes (β_a_) alter noticeably with the addition of **TZ1** and **TZ2** suggests that the corrosion reaction's mechanism is unchanged and that it is just impeded by the simple adsorption mode^48^. Also, no discernible change was seen for E_corr_ (roughly 16 mV), which is less than 85 mV, indicating that the two inhibitors behaved as mixed inhibitors that affected the anodic as well as cathodic processes^41^. The OCP values listed in Table 4 are comparable to the E_corr_ and show that the CS electrode potential shifted to a less negative value, significantly in **TZ1**, with increasing the inhibitor concentration. Indicating that the CS becomes less active toward dissolution reaction in inhibitor-containing acid compared to that in free acid. According to PP and OCP tests, **TZ1** is more effective to be used as a corrosion inhibitor than **TZ2**.

**Table 4:** Corrosion parameters of CS computed from PP method utilizing 1M HCl without and after addition of different concentrations of **TZ1** and **TZ2** at 25 °C.

Inhibitor	Conc, M	i_corr_ μA cm^−2^	− E_corr_, mVvs Ag/AgCl	− E_OCP_ mVvs Ag/AgCl	β_a_ mVdec^−1^	β_c_ mVdec^−1^	CR Mpy	Ө	%IE
Blank	1 M HCl	226.3	463	479	42.30	109.9	82.68	–	–
**TZ1**	1 × 10^−5^	69.63	450	476	81.50	141.2	25.43	0.692	69.2
	3 × 10^−5^	54.75	452	462	90.10	138.8	20.00	0.758	75.8
	5 × 10^−5^	49.25	463	459	83.20	165.9	17.99	0.782	78.2
	7 × 10^−5^	42.88	458	455	85.20	163.0	15.67	0.811	81.1
	9 × 10^−5^	31.38	447	449	100.5	130.5	11.46	0.861	86.1
**TZ2**	1 × 10^−5^	102.5	463	478	92.80	170.0	46.82	0.547	54.7
	3 × 10^−5^	99.63	458	477	78.10	150.5	45.49	0.560	56.0
	5 × 10^−5^	90.63	457	476	73.30	139.4	41.40	0.600	60.0
	7 × 10^−5^	78.25	455	475	80.70	134.6	35.76	0.654	65.4
	9 × 10^−5^	58.88	457	473	84.90	148.8	26.92	0.740	74.0

#### EIS measurements

**Figure 8:**
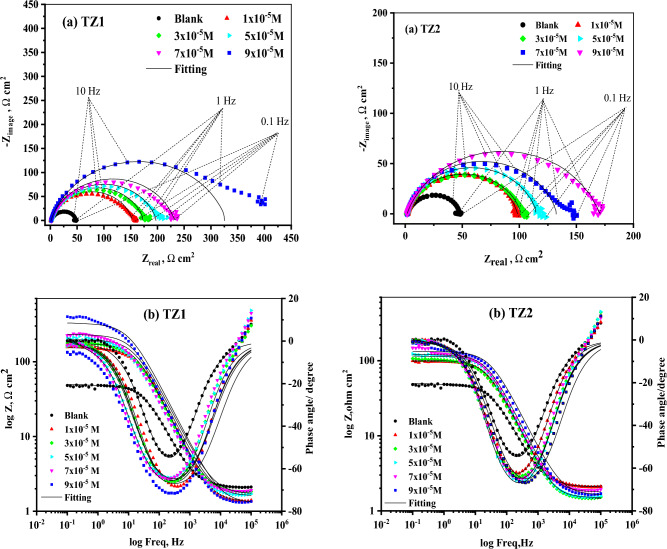
Nyquist (**a**) and Bode (**b**) plots for CS in 1M HCl and in the existence of various concentrations of **TZ1** and **TZ2** at 25°C.

**Figure 9:**
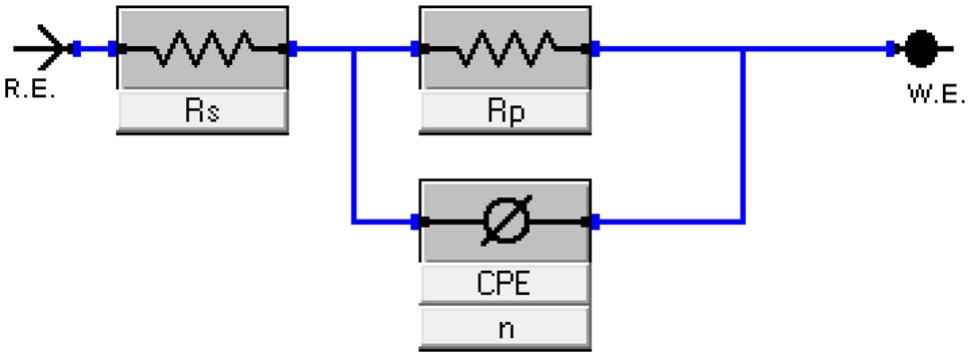
Electrical equivalent circuit to fit EIS data.

EIS is a useful technique for examining corrosion. EIS curves of CS in 1M HCl and in the existence of different concentrations of **TZ1** and **TZ2** at 25°C are shown in Fig. 8. These curves demonstrate that all of the produced Nyquist plots (Fig. 8a) are nearly semicircular and that their diameter increases as the inhibitor concentration rises. This shows that as the concentration of an inhibited substrate rises, so does its impedance. This finding demonstrates that the charge transfer process mostly controls CS corrosion in 1M HCl and in the existence of investigated inhibitors^49^. The imperfect circular shape of the capacitive loops deviates from the ideal shape, which is attributable to frequency dispersion brought on by surface roughness, the development of porous layers, dislocations, as well as surface inhomogeneities^50^. However, in Fig. 8a there is a noise observed at a low-frequency region in the EIS arc for all concentrations in both inhibitors. This could be attributed to heterogeneity at the electrode/electrolyte interface that comes from the release of a significant amount of the corrosion product as well as the interaction between the inhibitor active sites and the electrode surface. Figure 8b depicts the Bode plots for the two inhibitors, the impedance value increased with increasing the inhibitors concentration, and the larger impedance indicates that **TZ1** offers better protection for CS than **TZ2**^51^. Table 5 lists impedance parameters developed from fitting the data to the most appropriate electrical circuit (Fig. 9) including charge transfer resistance (R_ct_), double layer capacitance (C_dl_), and inhibition efficiency (%IE). It is clear that R_ct_ values rise as inhibitor concentrations rise while C_dl_ values fall. Also, it is observed that the diameters of the semi-circles for **TZ1** < **TZ2** confirm that **TZ1** is the most effective inhibitor. Adsorption of **TZ1** and **TZ2** at the metal/solution contact is responsible for this. The following Eq. (10) was used to determine the C_dl_ at the frequency f_max_^52^.

10 $${\text{C}}_{{{\text{dl}}}} = {1}/{2}\uppi \;{\text{f}}_{{{\text{max}}}} {\text{R}}_{{{\text{ct}}}}$$

**Table 5:** EIS data of CS in 1 M HCl and in the existence of various concentrations of **TZ1** and **TZ2** at 25 °C.

Inhibitor	Conc, M	R_ct_, (Ωcm^2^)	C_dl_, (μFcm^−2^)	Ө	% IE
Blank	1 M HCl	46.49	99.41	–	–
**TZ1**	1 × 10^−5^	152.0	55.73	0.694	69.4
	3 × 10^−5^	172.2	55.60	0.730	73.0
	5 × 10^−5^	196.8	50.71	0.764	76.4
	7 × 10^−5^	230.3	47.67	0.798	79.8
	9 × 10^−5^	327.6	45.60	0.858	85.8
**TZ2**	1 × 10^−5^	96.16	96.89	0.517	51.7
	3 × 10^−5^	100.0	95.58	0.535	53.5
	5 × 10^−5^	113.6	95.34	0.591	59.1
	7 × 10^−5^	131.3	81.06	0.646	64.6
	9 × 10^−5^	168.6	71.33	0.724	72.4

Owing to the growing surface coverage of inhibitors on the surface of CS, the %IE rises when inhibitor concentration is increased. The results of the EIS measurements are in agreement with those attained using PP and WL methods.

### Surface examinations

#### AFM analysis

**Figure 10:**
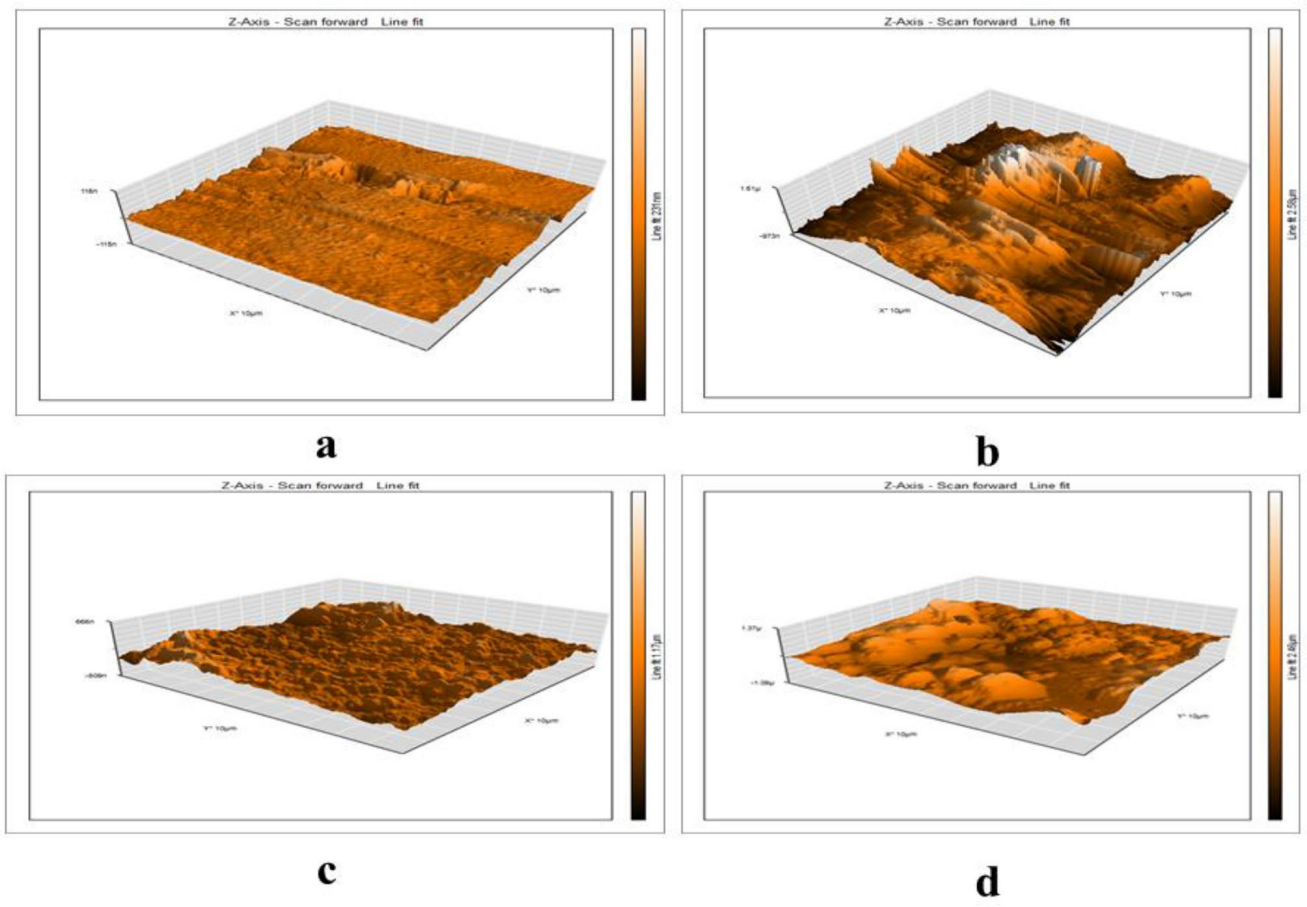
AFM images for CS surface: Polished CS (**a**); CS immersed in HCl solution (**b**); CS immersed in HCl solution containing (**TZ1**) (**c**); (**TZ2**) (**d**).

The surface morphology was investigated on the nanoscale by employing AFM for analyzing the major impact of **TZ1** and **TZ2** on the CS corrosion to establish the efficacy of the synthesized inhibitors as corrosion inhibitors^53,54^. Figure 10 shows three-dimensional AFM images for polished CS surfaces, surfaces immersed in 1M HCl, and surfaces with the existence of optimal inhibitors concentration (9 × 10^−5^ M) for 24 h. The average roughness of the polished CS surface was 6.91 nm (Fig. 10a), and caused by 1M HCl was 652.06 nm (Fig. 10b) but the existence of 9 × 10^−5^ M of **TZ1** and **TZ2** in 1M HCl reduced the average roughness of CS surface to 57.92 and 157.05 nm, respectively, as displayed in Fig. 10c and d. This difference in average roughness values provides information about the effective adsorption of **TZ1** and **TZ2** on the CS surface and creates a shielding layer that reduces the attack caused by the 1M HCl^55^. These roughness values corroborate the results of both chemical and electrochemical procedures.

#### XPS analysis

**Figure 11:**
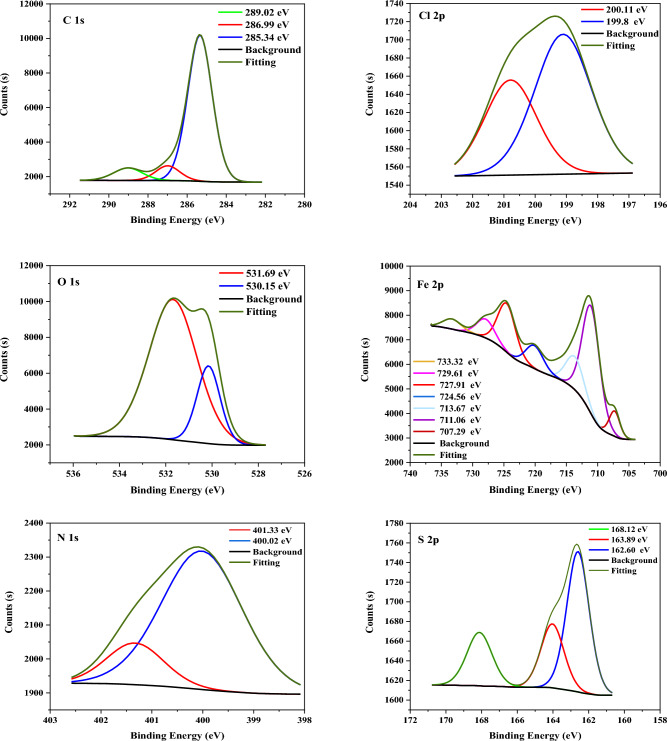
XPS spectra for CS utilizing 1M HCl with 9 × 10^−5^ M of **TZ1**.

**Figure 12:**
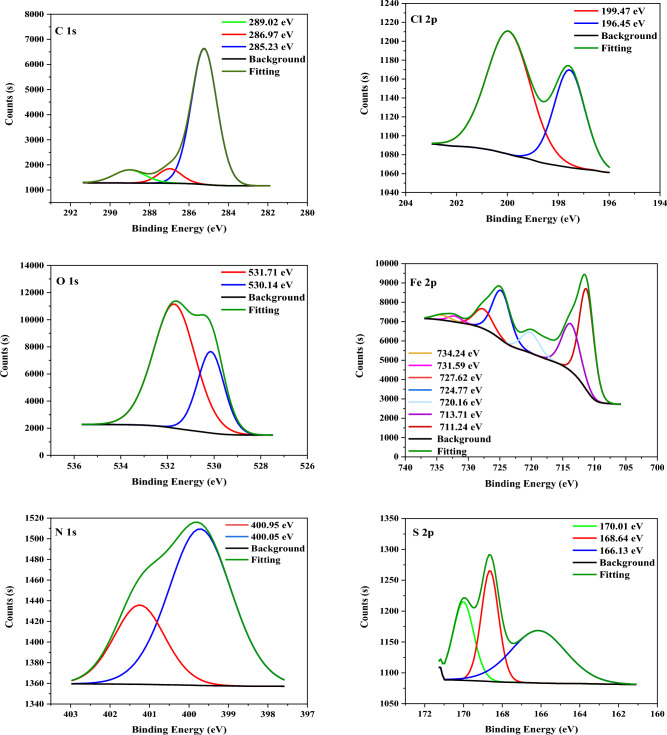
XPS spectra for CS utilizing 1M HCl with 9 × 10^−5^ M of **TZ2**.

The structure of **TZ1** and **TZ2** molecules and chemical bonds were identified and described using XPS analysis, which was also used to demonstrate that the molecules had adhered to the CS surface. Figures 11 and 12 display the XPS spectra for CS surfaces subjected to 1 M HCl with the inclusion of **TZ1** and **TZ2** molecules. The obtained spectra were composed of C 1s, Cl 2p, Fe 2p, O 1s, N 1s, and S 2p. The appearance of N 1s and S 2p peaks supports the adsorption of **TZ1** and **TZ2** molecules on the CS surface. Table 6 displays the binding energies (BE, eV) and the corresponding assignment of each peak component. In the C 1s involved spectra, three peaks for **TZ1** and **TZ2** were seen (Figs. 11, 12). The first peak, which occurs at 285.34, 285.23 eV, could be due to C–H, C–C, and C=C bonds^56^. The second peak, which occurs at 286.99, 286.97 eV, could be related to C–N and C–S bonds^57^. The final peak, which occurs at 289.02 eV, could be related to O–C=O and C=N for **TZ1**^58,59^ and C=N for **TZ2**, which are likely found in the structure of **TZ1** and **TZ2** molecules, verifying their adsorption. Cl 2p spectra (Figs. 11, 12) display two peaks for Cl 2p_3/2_ at 199.80, 196.47 eV and Cl 2p_1/2_ at 200.11, 199.47 eV, respectively^60^. The spectra of O 1s contain two peaks (Figs. 11, 12). The first peak, which is assigned to O^2−^ and has a binding energy of 530.15, 530.14 eV, could be related to the connection between an oxygen atom and (Fe^3+^in the Fe_2_O_3_ and/or Fe_3_O_4_ oxide), while the last located at 531.69, 531.71 eV could be attributed to OH^-^, due to the existence of hydrous iron oxides, such as FeOOH^57^. The XPS spectra of Fe 2p display seven peaks (Figs. 11, 12) at 707.29, 711.24 eV for metallic iron, 711.06, 713.71 eV for Fe^3+^, 713.67, 720.16 eV for Fe 2p_3/2_ of Fe^2+^, 724.56, 724.77 eV for satellite of Fe^3+^, 727.91, 727.62 eV for Fe 2p_1/2_ of Fe^2+^, 729.61, 731.59 eV for Fe 2p_1/2_ of Fe^3+^and 733.32, 734.24 eV for Fe 2p_1/2_ of Fe^2+^^61^. N 1 s spectra (Figs. 11, 12) depict two peaks at 400.02, 400.05 eV and 401.33, 400.95 eV which may be caused by N–Fe, N–N, and C=N–N bond in the triazole ring, respectively^62,63^. Three peaks were observed in the S 2p spectra (Figs. 11, 12), the peaks at 162.60 eV and 163.89, 166.13 eV could be related to the S–C bond from the thiol group (C–SH). The last peaks located at 168.12, 168.64, and 170.01 eV could be related to S–Fe bond^57^. As a result, the adsorption of **TZ1** and **TZ2** using 1 M HCl on the CS surface was verified by XPS measurements.

**Table 6:** Binding energies (eV), and their assignments observed for CS surface treated with 9 × 10^−5^ M of **TZ1** and **TZ2** in 1M HCl.

Core element	1M HCl + **TZ1**		1M HCl + **TZ2**	
	BE, eV	Assignments	BE, eV	Assignments
C 1 s	285.34	C–H, C–C, and C=C bonds	285.23	C–H, C–C, and C=C bonds
	286.99	C–N and C–S bonds	286.97	C–N and C–S bonds
	289.02	O–C=O and C=N	289.02	C=N
Cl 2p	199.80	Cl 2p_3/2_	196.47	Cl 2p_3/2_
	200.11	Cl 2p_1/2_	199.47	Cl 2p_1/2_
O 1 s	530.15	Fe_2_O_3_ and/or Fe_3_O_4_ oxide	530.14	Fe_2_O_3_ and/or Fe_3_O_4_ oxide
	531.69	FeOOH	531.71	FeOOH
Fe 2p	707.29	metallic iron	711.24	metallic iron
	711.06	Fe^3+^	713.71	Fe^3+^
	713.67	Fe 2p_3/2_ of Fe^2+^	720.16	Fe 2p_3/2_ of Fe^2+^
	724.56	satellite of Fe^3+^	724.77	satellite of Fe^3+^
	727.91	Fe 2p_1/2_ of Fe^2+^	727.62	Fe 2p_1/2_ of Fe^2+^
	729.61	Fe 2p_1/2_ of Fe^3+^	731.59	Fe 2p_1/2_ of Fe^3+^
	733.32	Fe 2p_1/2_ of Fe^2+^	734.24	Fe 2p_1/2_ of Fe^2+^
N 1 s	400.02	N–Fe, N–N	400.05	N–Fe, N–N
	401.33	C=N–N bond	400.95	C=N–N bond
S 2p	162.60 ,163.89	S–C bond	166.13	S–C bond
	168.12	S–Fe bond	168.64,170.01	S–Fe bond

### Analysis of test solution (UV–Visible spectroscopy)

**Figure 13:**
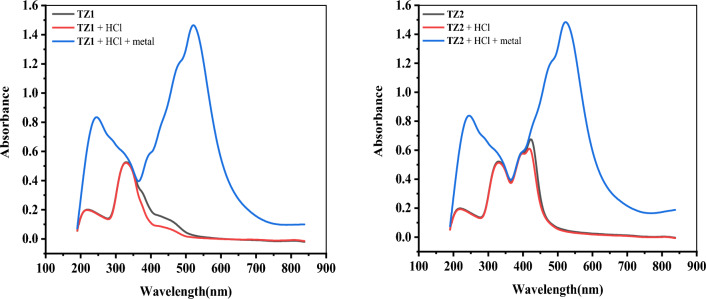
UV–Visible spectra for **TZ1** and **TZ2** (black color), 1M HCl solution containing **TZ1** and **TZ2** before (red color) and after (blue color) dipping CS for 48 h at room temperature.

UV–visible spectroscopy measurements were done for both inhibitors in three different solutions: inhibitor only, inhibitor + HCl, and inhibitor + HCl + CS immersed for 48 h at 25 °C. The concentration of inhibitor and HCl were kept at 9 × 10^−5^ M and 1.0 M, respectively in all three mixtures. According to the spectra (Fig. 13), **TZ1** and **TZ2** showed peaks at (220 nm, 330 nm) and (219nm, 330 nm, 423 nm), respectively, which may be attributed to π–π* and n–π* transitions^64^. The spectra of the inhibitors in 1 M HCl before CS immersion shows a small shift(insignificant) in wavelength but after the immersion of CS, the spectra of **TZ1** and **TZ2** reveal some change in the adsorption bands. The spectrum of **TZ1** showed two bands at 245 nm and 521nm, and the spectrum of **TZ2** also indicates two well-distinguished bands at 245 nm and 523 nm. According to the literature^65^, the change in the position of the absorption maximum (λ_max_) and/or the variation of the absorbance value suggest the formation of a complex between the triazole compounds and the Fe^2+^ ions in the solution. Therefore, the obtained results from the UV–visible spectral analysis of the inhibitors before and after CS immersion demonstrate that the thiol, (C=N–N), double bonds of triazole ring, coumarin ring, and fluorene are primarily involved in the adsorption of these inhibitors on CS surface. This suggests that triazole derivatives can form a complex with iron atoms according to a donor–acceptor mechanism, confirming the chemisorption process of **TZ1** and **TZ2** on CS surface^64^.

### Antibacterial activity

The result of antibacterial activity with zones of inhibition measured in millimeters is as shown in Table 7. **TZ1** showed good inhibitory effect against *E. coli* and *B. subtilis* with inhibition zones of 15 and 17 mm, respectively while **TZ2** exhibited good inhibitory effect against *E. coli* (17 mm) but no activity against *B. subtilis*. Finally, the antibiotic sensitivity of Ciprofloxacin (CIP) showed the highest inhibitory effect against the two micro-organisms with inhibition zone of 39 mm for *E. coli* and 29 mm for *B. subtilis*. **TZ1** and **TZ2** have antibacterial activity which demonstrates their environmentally friendly and anti-toxic nature.

**Table 7:** The results of antibacterial activity with zones of inhibition.

Samples	Zones of inhibition (mm)	
	*E. coli*	*B. subtilis*
**TZ1**	15 mm	17 mm
**TZ2**	17 mm	− ve
Control (DMSO)	− ve	− ve
Ciprofloxacin (CIP)	39 mm	29 mm

### DFT studies

**Figure 14:**
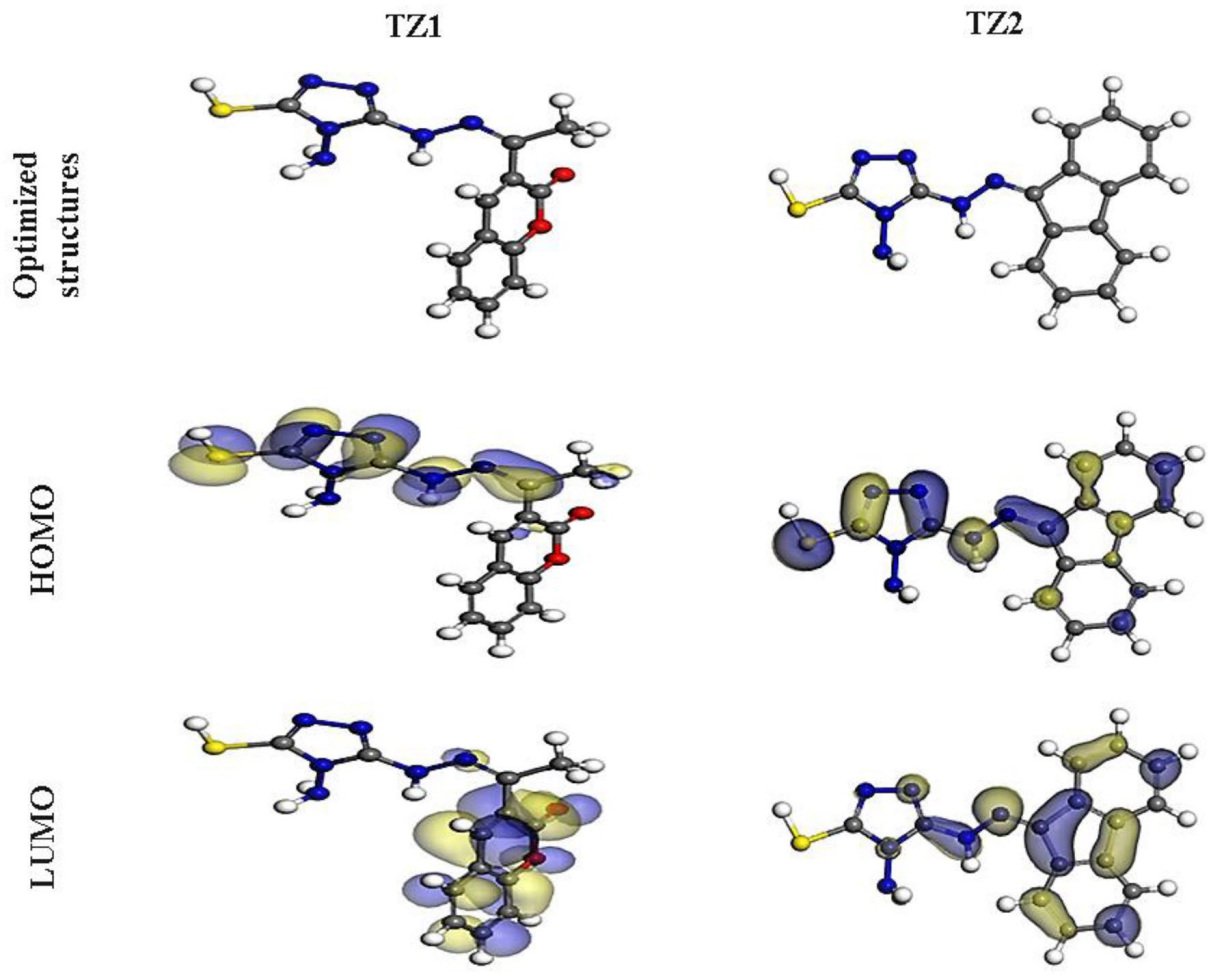
The optimized molecular structures, HOMO and LUMO of **TZ1** and **TZ2** utilizing DMol^3^ module.

**Figure 15:**
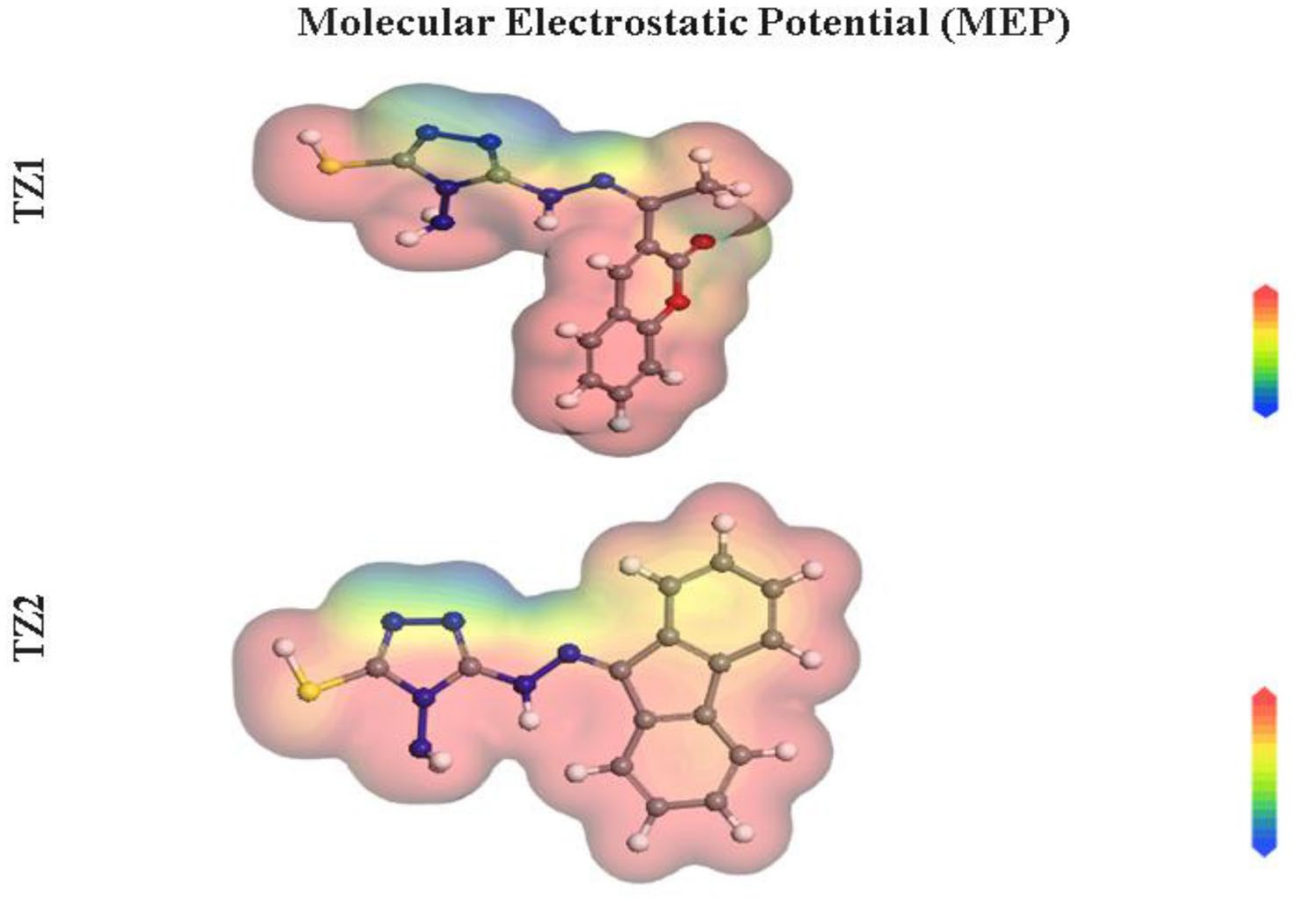
Graphical presentation of the MEP of **TZ1** and **TZ2** utilizing DMol^3^ module.

To investigate the chemical reactivity and determine the established association with the experimentally achieved inhibitory efficacy, DFT calculations are applied. Figure 14 depicts the optimized structures, HOMO and LUMO distributions, and the linked theoretical parameters for **TZ1** and **TZ2** are summarized in Table 8. According to the FMO theory, HOMO, as well as LUMO energies, specify the capacity of donor or acceptor interactions carried out at the surface of inhibitor/metal^66^. An inhibitor molecule with high E_HOMO_ and low E_LUMO_ values performs better in inhibiting corrosion. In contrast to the **TZ2** molecule (E_HOMO_ = − 4.65 eV), the **TZ1** molecule has a maximal E_HOMO_ value = − 4.63 eV, as perceived in Table 8. According to Fig. 14, it is clear that the HOMO level localized on the triazole ring and hydrazone moieties for **TZ1** and **TZ2**, indicating that the sulfur, as well as nitrogen atoms, are the favored sites for electrophilic attacks on the CS surface. Such interpretations promote the capability of the inhibitor to adsorb on the CS surface, causing an improvement in the performance of the protection, which was consistent with the experimental findings. Conversely, the E_LUMO_ value of the **TZ1** molecule is − 2.61eV, which is lower than that of the **TZ2** molecule (− 2.42 eV). The lower E_LUMO_ value for the **TZ1** molecule indicates that it has excellent protective capabilities than **TZ2**, which agrees with the experimental findings. Likewise, the ∆E (energy gap) is a crucial parameter to enhance the inhibitor's ability to resist corrosion, which improves as the ∆E value decreases^67^. According to Table 8, **TZ1** is more likely to be adsorbed on the surface of CS because it has a lower ∆E value (2.02 eV) than **TZ2** (2.23 eV). The low values of electronegativity (χ) suggest a high potential reactivity of the **TZ1** and **TZ2** molecules to provide electrons to the CS surface^68^. Additionally, the hardness (η) as well as softness (σ) of a molecule may be utilized to establish its reactivity and stability. The reactivity of soft molecules is higher than hard molecules because they provide electrons more readily to the CS surface via adsorption, making them effective corrosion inhibitors^69^. Table 8 shows that **TZ1** has higher σ value and lower η value than **TZ2**, indicating easy electron donation to CS surface and excellent inhibitory proficiency for **TZ1** molecule. Also, the ∆N and ∆E_back-donation_ are key factors in determining the inhibitor's capacity for either donating or receiving electrons. As a result, if the ∆N values are greater than zero, the inhibitor can transfer electrons to iron surface, and if they are less than zero, metal atoms can donate electrons to the inhibitor molecule (i.e., back-donation)^70,71^. The ∆N values of **TZ1** and **TZ2** are more than zero as reported in Table 8, showing that the inhibitors are able to transfer electrons to the iron surface. Additionally, when η is greater than 0, the ∆E_back-donation_ will be less than 0. This is because an electron that is transferred to an inhibitor molecule is followed by a back donation from the inhibitor molecule, which is dynamically desired^72^. The ∆E_back-donation_ values for **TZ1** and **TZ2** molecules in Table 8 are negative, revealing that back-donation is favored for the inhibitor molecules and creates a strong bond^73^. Furthermore, the dipole moment is a crucial marker that aids in predicting the pathway of corrosion inhibition^74^. The improvement in deformation energy and augmentation of molecule adsorption on the steel contact are both made possible by the increase in dipole moment. As a result, an increase in dipole moment leads to an improvement in corrosion prevention effectiveness^75^. **TZ1** molecule has a higher dipole moment value (12.60 Debye) than **TZ2** molecule (10.06 Debye), as shown in Table 8, which supports its stronger propensity to adsorb onto the CS surface. Furthermore, there is a clear link among the propensity of **TZ1** and **TZ2** molecules to shield the CS surface from acidic media and their molecular surface area. The increased protective capability is correlated with larger size of molecular structure as the contact area among **TZ1** and **TZ2** molecules and CS surface increases^76^. Table 8 indicates that **TZ1** has the highest molecular surface area (321.19 Å^2^) and therefore greater inhibitory efficacy when compared to **TZ2** (314.47 Å^2^). Additionally, the Dmol^3^ module's ability to estimate molecular electrostatic potential (MEP) mapping to look into the active sites of the inhibitors under investigation. MEP mapping is a 3D visual representation that aims to identify a molecule's net electrostatic impact based on its general charge distribution^67^. The highest electron density area is represented by the red colors in the MEP maps shown in Fig. 15, in which the MEP is highly negative (nucleophilic reaction). However, the strongest positive area (electrophilic reaction) is represented by the blue colors^60^. The greatest negative regions (red colors) in Fig. 15 are mostly over free nitrogen, sulphur, and oxygen atoms for **TZ1** but over free nitrogen, sulphur, and aromatic rings for **TZ2**, whereas the most positive regions (i.e., blue colors) over allocated nitrogenous atom of triazole ring as hybridization of lone pairs allocated on the nitrogenous atoms of triazole at positions (1,2) is sp^2^ orbital which allocate in the same plane of the ring and does not overlap with other p orbitals of the ring. The highest potential places for interactions with the CS surface could be those with high electron densities in **TZ** molecules.

**Table 8:** The computed quantum chemical parameters for **TZ1** and **TZ2** molecules.

Quantum parameters	**TZ1**	**TZ2**
E_HOMO_, eV	− 4.63	− 4.65
E_LUMO_, eV	− 2.61	− 2.42
∆E = E_LUMO_ − E_HOMO_, eV	2.02	2.23
I, eV	4.63	4.65
A, eV	2.61	2.42
χ, eV	3.62	3.54
η, eV	1.01	1.11
σ, eV	0.99	0.90
∆N, eV	0.594	0.577
∆E_back-donation_, eV	− 0.25	− 0.28
Dipole moment value, debye	12.60	10.06
Molecular surface area, Å^2^	321.19	314.47

### MC simulations

**Figure 16:**
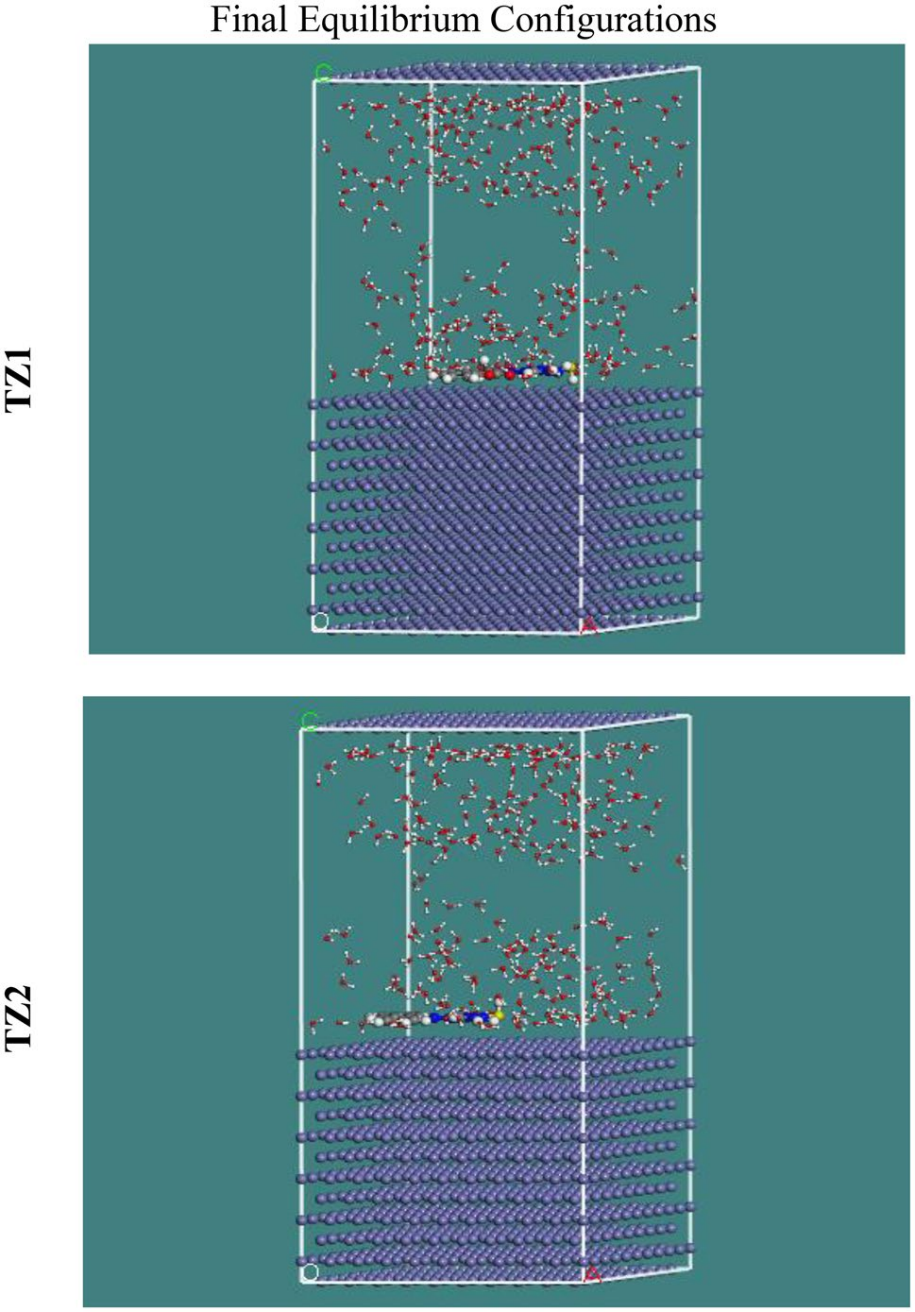
The appropriate configuration for adsorption of **TZ1** and **TZ2** on Fe (11 0) utilizing adsorption locator module.

The interactions between **TZ1** and **TZ2** molecules and the CS surface as well as the adsorption mechanism were visualized using MC simulations. The most probable adsorption configurations for **TZ1** and **TZ2** molecules on the CS are depicted in Fig. 16. This was made possible via the adsorption locator module, which displays smooth disposition as well as offers an enhancement in the adsorption with the most surface coverage^77^. Table 9 lists the data that was determined via MC simulations. The Table summarizes the adsorption energies of **TZ1** and **TZ2** (both unrelaxed and relaxed) before and after the geometry optimization process. It is found that **TZ1** (− 3061.79 kcal mol^−1^) has a higher negative value of adsorption energy than **TZ2** (− 3034.04 kcal mol^−1^), implying robust adsorption of **TZ1** on the CS surface creating a fixed adsorbed film, which is consistent with the experimental data^77^. The dE_ads_/dN_i_ values describe the metal-adsorbate configuration's energy if one of the adsorbates is eliminated^78^. **TZ1** molecules have superior adsorption than **TZ2** molecules, as evidenced by the fact that their dE_ads_/dN_i_ value is higher (− 194.09 kcal mol^−1^) than that of **TZ2** molecules (− 187.14 kcal mol^−1^). Furthermore, the dE_ads_/dN_i_ value for water is about − 7.36 kcal mol^−1^, which is low when linked to the **TZ1** and **TZ2** values, indicating that these two investigated inhibitors were adsorbed more strongly than water molecules on the CS surface, supporting the replacement of water molecules with **TZ1** and **TZ2** molecules. Thus, it can be summarized that these MC results correspond well with the quantum chemical calculations as well as the experimental data.

**Table 9:** Data calculated utilizing MC simulations for the adsorption **TZ1** and **TZ2** on Fe (1 1 0).

Structures	Adsorption energy/Kcal mol^−1^	Rigid adsorption energy/ kcal mol^*−*1^	Deformation energy/ kcal mol^*−*1^	dE_ads_/dNi: Inhibitor kcal mol^*−*1^	dE_ads_/dNi: Water kcal mol^*−*1^
Fe (1 1 0) **TZ1** Water	− 3061.79	− 3208.50	146.71	− 194.09	− 6.95
Fe (1 1 0) **TZ2** Water	− 3034.04	− 3183.56	149.52	− 187.14	− 7.76

### Corrosion inhibition mechanism

**Figure 17:**
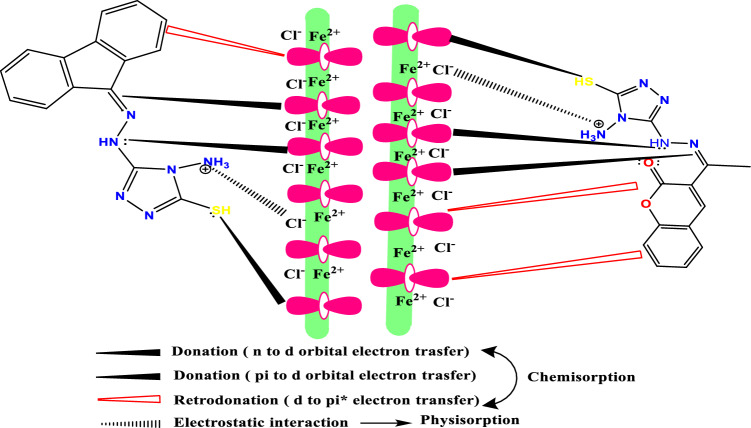
Possible adsorption mechanism of **TZ1** and **TZ2** on CS surface in HCl solution.

It is possible to propose a schematic mechanism for the interaction of tested triazole derivatives with CS surface based on the data we obtained in this work, as displayed in Fig. 17. **TZ1** and **TZ2** molecules can be chemically adsorbed through acceptor–donor interactions^79^. The presence of many functional groups and heteroatoms in addition to electronic pairs as well as pi bonds can ensure the donation of the electron to the vacant d-orbitals of iron and thus form strong bonds (chemisorption) with the CS surface. However, inter-electronic repulsions result from the transfer of electrons from the inhibitor to vacant d-orbitals of metal. The filled d-orbitals of the metal atoms will transfer electrons in the reverse order to the vacant antibonding molecular orbitals of **TZ1** and **TZ2** by retro-donation to avoid these repulsions, therefore enhancing the adsorption on the metal surface as it is reported that more electron donation causes an increase in retro-donation, and therefore donation and retro-donation reinforce each other via synergism^80^. In the HCl solution, **TZ1** and **TZ2** can also be protonated due to the presence of an NH_2_ group with higher electron density, which promotes the electrostatic interaction with the negatively charged metal surface created by Cl- ions (physisorption)^81^.

According to the collected theoretical data, **TZ1** was more efficient than **TZ2** because **TZ1** has the highest molecular surface area so it covers a larger area from the CS surface, highest dipole moment, has the highest adsorption energy, the highest softness value (more reactive) and lowest energy gap. As a result, **TZ1** can donate more electron pairs to the d-orbital of iron than **TZ2**. These electrons will be accumulated on the CS surface and followed by a retro-donation from the filled d-orbital of iron to Π* (C=C, C=O), resulting in the stronger adsorption of **TZ1** on the CS surface than **TZ2** (retro-donation from d-orbital to Π* (C=C)).

### Comparative studies with previous reports

In this study, two novel 1,2,4-triazole derivatives were synthesized, and their effectiveness as corrosion inhibitors for carbon steel (CS) in a 1.0 M HCl solution was evaluated using various techniques. Table 10 illustrates the inhibitory effect of (**TZ1** and **TZ2**) and other 1,2,4-triazole compounds for CS corrosion in an acidic medium. As represented in Table 10, **TZ1** and **TZ2** inhibitors exhibited comparable corrosion inhibition efficiencies when compared to other 1,2,4-triazole derivatives. Notably, the used concentrations of **TZ1** and **TZ2** were more than tenfold lower than those of other derivatives listed in Table 10, which makes these current inhibitors cost-effective and favorable for use. Furthermore, the effectiveness of **TZ1** and **TZ2** in inhibiting CS corrosion increases with rising temperatures.

**Table 10:** Comparative studies with previously studied 1,2,4-triazole derivatives in literature.

Inhibitor	Corrosive medium	Conc. of inhibitor	% IE		Sample	References
			PP	EIS		
(*Z)*-4-((4-methoxybenzylidene)amino)-5-methyl-2,4-dihydro-3*H*-1,2,4-triazole-3-thione (2C)	1M HCl	10^−3^ M	83.00	86.00	CS	^82^
(Z)-4-((2,4-dihydroxybenzylidene) amino)-5-methy-2,4- dihydro-3H-1,2,4-triazole-3-thione	1M HCl	10^−3^ M	81.37	81.67	Mild steel	^83^
3,5-Bis(4-methoxyphenyl)-4-amino-1,2,4-triazole (T1)	2M H_3_PO_4_	10^−3^ M	85.72	86.81	Mild steel	^84^
3,5-Bis(4-chlorophenyl)-4-amino-1,2,4-triazole (T2)			83.49	86.20		
(Z)-4-((2-bromobenzylidene) amino)-5-methyl-2-4-dihydro-3H-1,2,4-triazole-3-thione (2i)	1M HCl	10^−3^ M	83.66	89.51	Mild steel	^85^
(Z)-4-((3-bromobenzylidene) amino)-5-methyl-2-4-dihydro-3H-1,2,4 -triazole-3-thione (2I)			82.84	84.50		
(3-Bromo-4-fluoro-benzylidene)-[1,2,4]triazol-4-yl-amine (BFBT)	0.5M HCl	3.2 mM	91.21	85.05	Mild steel	^86^
(4-trifluoromethyl-benzylidene)-[1,2,4]triazol-4-yl-amine (TMBT)			85.17	80.09		
(2-Fluoro-4-nitro-benzylidene)-[1,2,4]triazol-4-yl-amine (FNBT)			84.36	72.83		
3,5-Bis(4-tolyl)-4-amino-1,2,4-triazole (K1)	2M H_3_PO_4_	10^−3^ M	79.83	80.00	Mild steel	^87^
3,5-Bis(3,4-dimethoxyphenyl)-4-amino-1,2,4-triazole (K2)			83.32	86.35		
(*Z*)-3-(1-(2-(4-amino-5-mercapto-4*H*-1,2,4-triazol-3-yl)hydrazono)ethyl)-2*H*-chromen-2-one (**TZ1**)	1M HCl	9 × 10^−5^ M	86.10	85.80	CS	Our work
5-(2-(9*H*-fluoren-9-ylidene)hydrazineyl)-4-amino-4*H*-1,2,4-triazole-3-thiol (**TZ2**)			74.00	72.40		

## Conclusion

1. Two novel 4-amino-5-hydrazineyl-4*H*-1,2,4-triazole-3-thiol derivatives (**TZ1** and **TZ2**) were synthesized, characterized, and then were tested as corrosion inhibitors for CS in 1M HCl solution and exhibited a very good inhibition via formation of a protective layer on CS surface.
2. The inhibition efficiency of these derivatives increases with increasing concentration and, with temperature increasing according to WL measurements, which reached 93.7% and 84.5% at 45°C in the presence of the optimum concentration (9 × 10^−5^ M) for **TZ1** and **TZ2**, respectively.
3. These derivatives were adsorbed onto CS in 1M HCl solution according to the Langmuir isotherm. The negative values of ∆G°_ads_ showed that the adsorption process of the **TZ1** and **TZ2** is spontaneous. The ∆G°_ads_ values suggested that the adsorption process of both inhibitors on the CS surface is mixed physical and chemical adsorption with a remarkable predominance of chemisorption, especially in the case of **TZ1**. In addition, the values of ∆G°_ads_ become more negative with increasing temperature, confirming that the chemisorption process is favored at high temperatures.
4. The values of E_a_* obtained in the existence of **TZ1** and **TZ2** were lower than the blank solution, which supports the chemical adsorption hypothesis.
5. According to the PP data, the shift in the corrosion potential to more positive values (roughly 16 mV), which is less than 85 mV, as a result, **TZ1** and **TZ2** acted as mixed inhibitors.
6. The EIS data indicates that the addition of **TZ1** and **TZ2** to the test solutions causes a decrease in C_dl_ and an increase in R_ct_ values compared to the blank solution, demonstrating the adsorption of inhibitor molecules on the CS surface.
7. The inhibition efficiency outcomes obtained from the WL measurements agreed well with the PP and EIS techniques.
8. From AFM and XPS data for surface analysis, it was confirmed that the tested inhibitors were well linked to the CS surface. The complex formation between ferrous ions and the investigated inhibitors was proved by UV–visible spectroscopy. **TZ1** showed good antibacterial activity against *E. coli* and *B. subtilis*. while **TZ2** showed higher antibacterial activity against *E. coli* and has no effect on *B. subtilis*. Therefore, the **TZ1** and **TZ2** can be used as inhibitors for CS corrosion in industrial applications.
9. The E_Homo_ (eV) and negative adsorption energies (Kcal mol^*−*1^) values have been demonstrated to be higher for **TZ1** than **TZ2**, indicating that **TZ1** is the most potent inhibitor based on DFT and MC simulations, respectively, which supports the findings of the experimental techniques.

### Supplementary Information

Supplementary Information.

## Data Availability

All data generated or analysed during this study are included in this published article (and its Supplementary Information files).
