# Supplementary material for: Novel synthesized triazole derivatives as effective corrosion inhibitors for carbon steel in 1M HCl solution: experimental and computational studies
Source: Sci Rep. 2023 Dec 13;13:22180. doi: 10.1038/s41598-023-49468-5 (PMC10719362; doi:10.1038/s41598-023-49468-5)
Supplement: Supplementary file 1 — Supplementary Information. [file 41598_2023_49468_MOESM1_ESM.docx]

Novel synthesized triazole derivatives as effective corrosion inhibitors for carbon steel in 1M HCl solution: experimental and computational studies

Kamelia Belal^1^, A.H. El-Askalany^1^, Eslam A Ghaith^1^, Ahmed Fathi Salem Molouk^*1,2^

^1^Department of Chemistry, Faculty of Science, Mansoura University, Mansoura 35516, Egypt

^2^Faculty of Science, New Mansoura University, New Mansoura City, Egypt

^*^ Correspondence author.

E-mail address: molouk82@mans.edu.eg

Supplementary Material

Figure caption

**S1**. ^1^H-NMR spectrum of compound **TZ1**

**S2**. ^1^H-NMR spectrum of D_2_O test for compound **TZ1**

**S3**. ^13^C-NMR spectrum of compound **TZ1**

**S4**. ^1^H-NMR spectrum of compound **TZ2**

**S5**. ^1^H-NMR spectrum of D_2_O test for compound **TZ2**

**S6**. ^13^C-NMR spectrum of compound **TZ2**

**S7.** Different adsorption isotherms of **TZ1** and **TZ2** for the corrosion of CS in 1 M HCl at 25℃.

Table caption

**Table S1.** Regression coefficient, slope, and intercept for different adsorption isotherm of **TZ1** and **TZ2**.


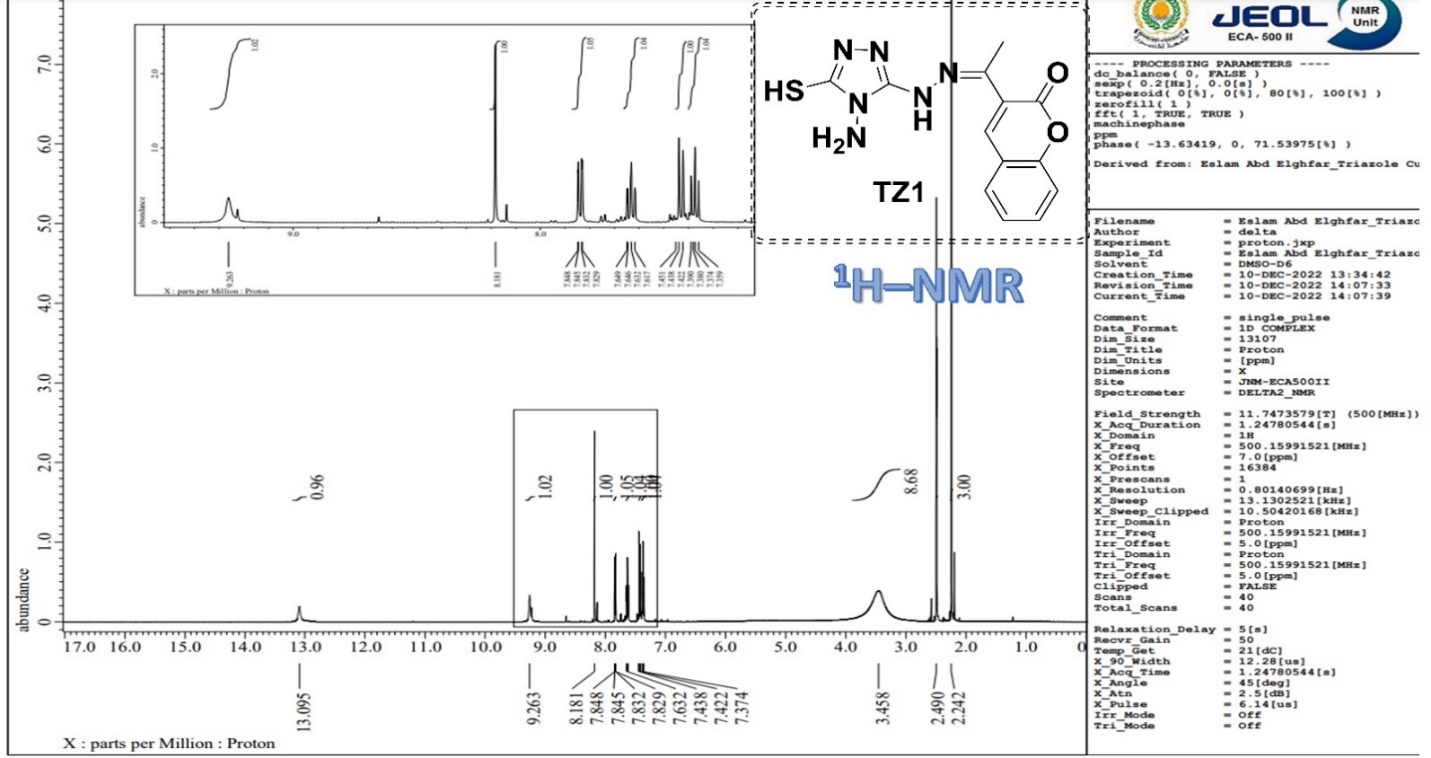


**S1**. ^1^H-NMR spectrum of compound **TZ1**


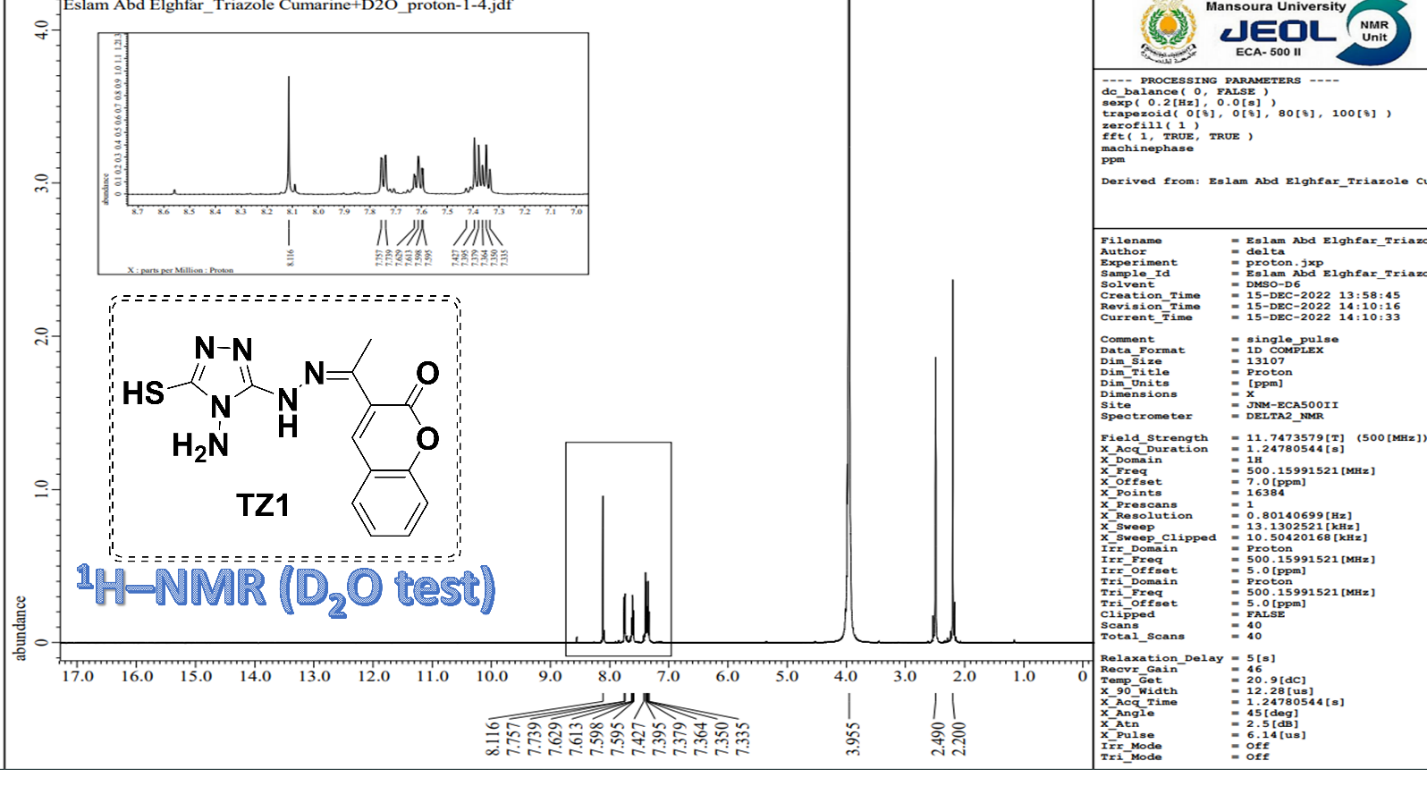
 **S2**. ^1^H-NMR spectrum of D_2_O test for compound **TZ1**


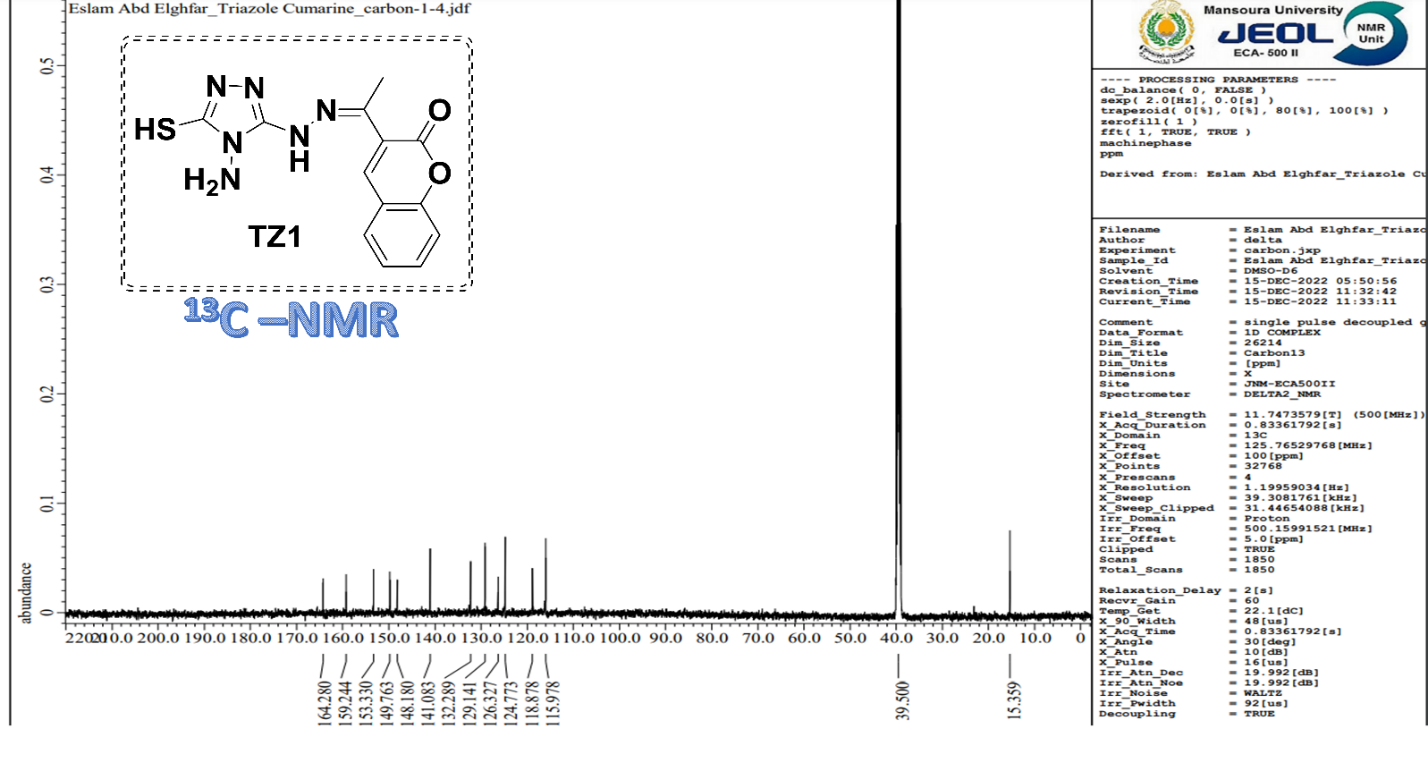
**S3**. ^13^C-NMR spectrum of compound **TZ1**


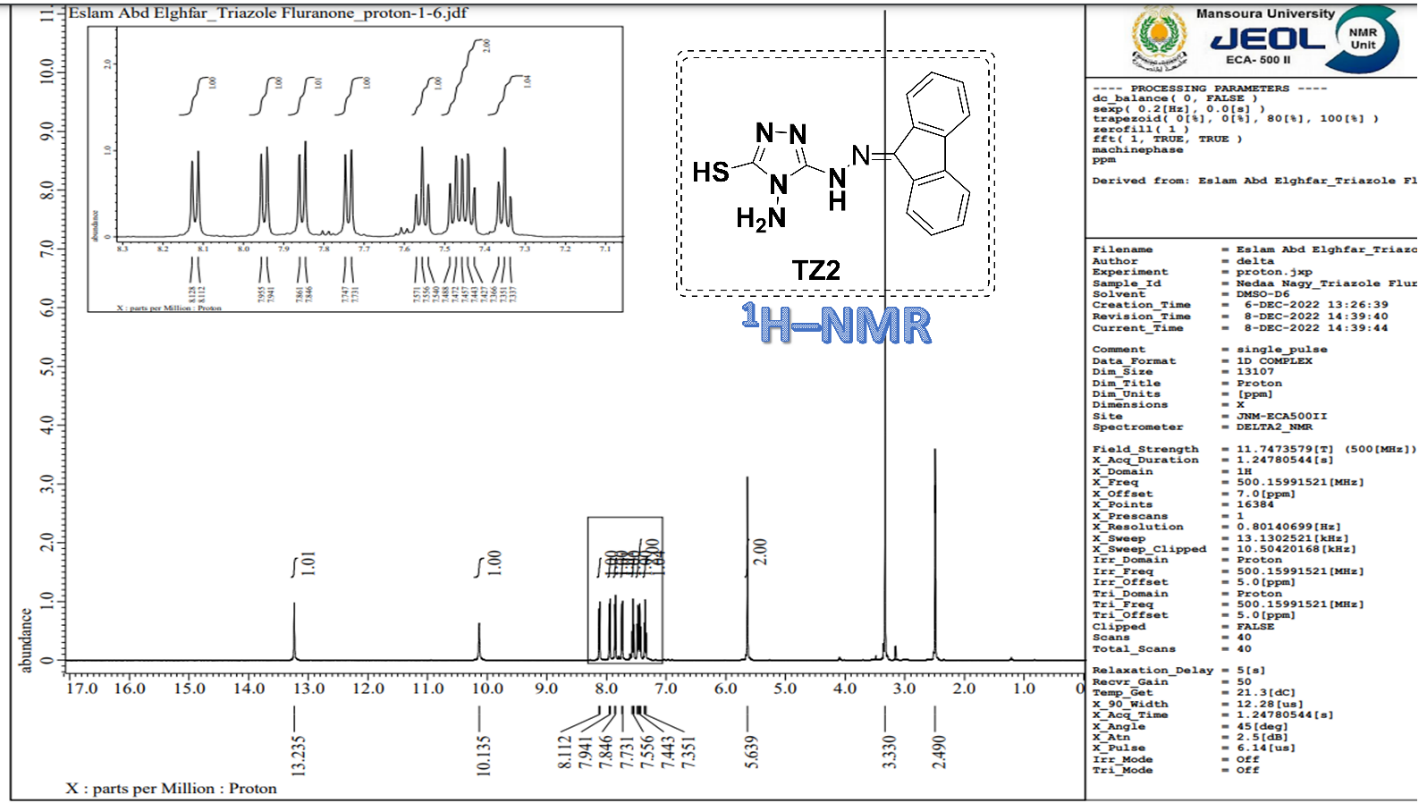
**S4**. ^1^H-NMR spectrum of compound **TZ2**


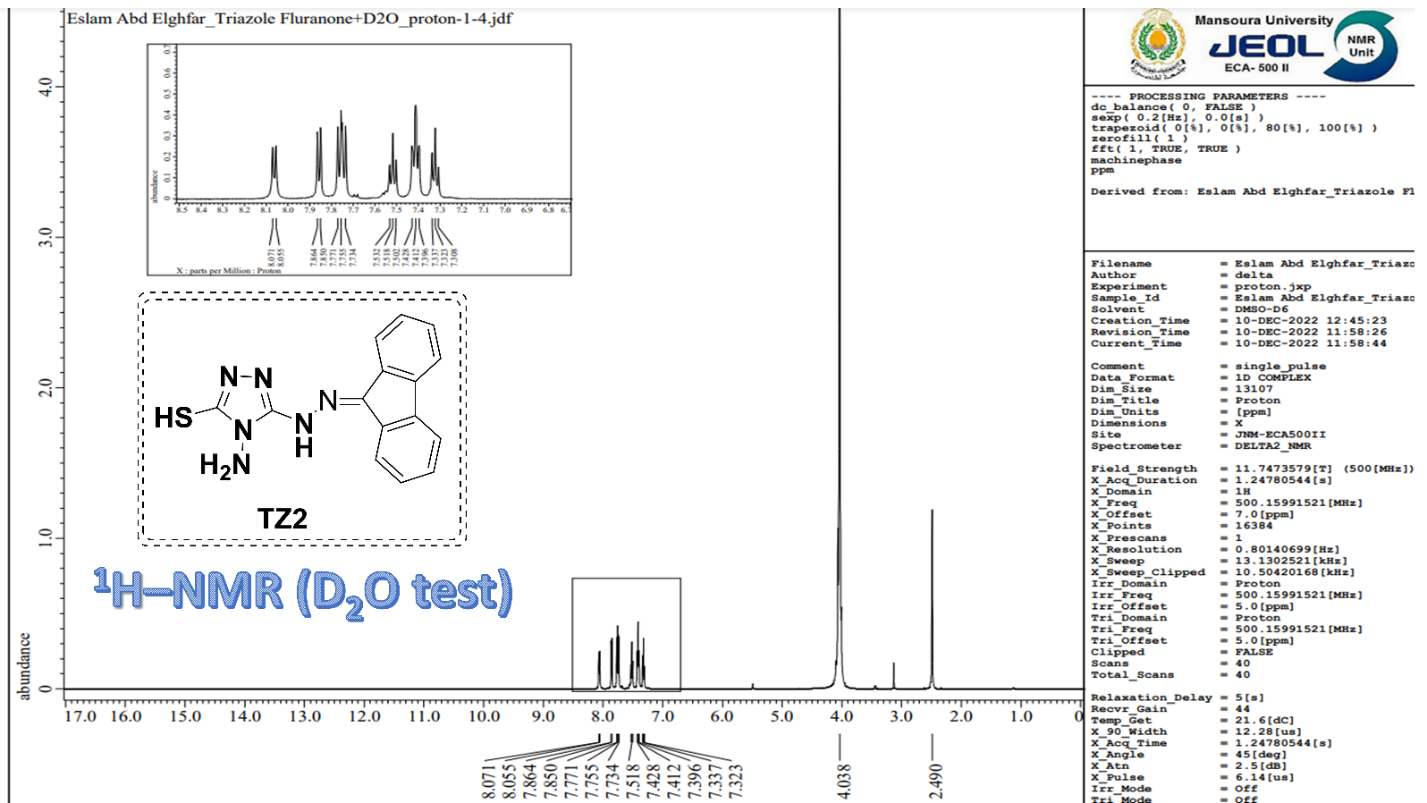
**S5**. ^1^H-NMR spectrum of D_2_O test for compound **TZ2**


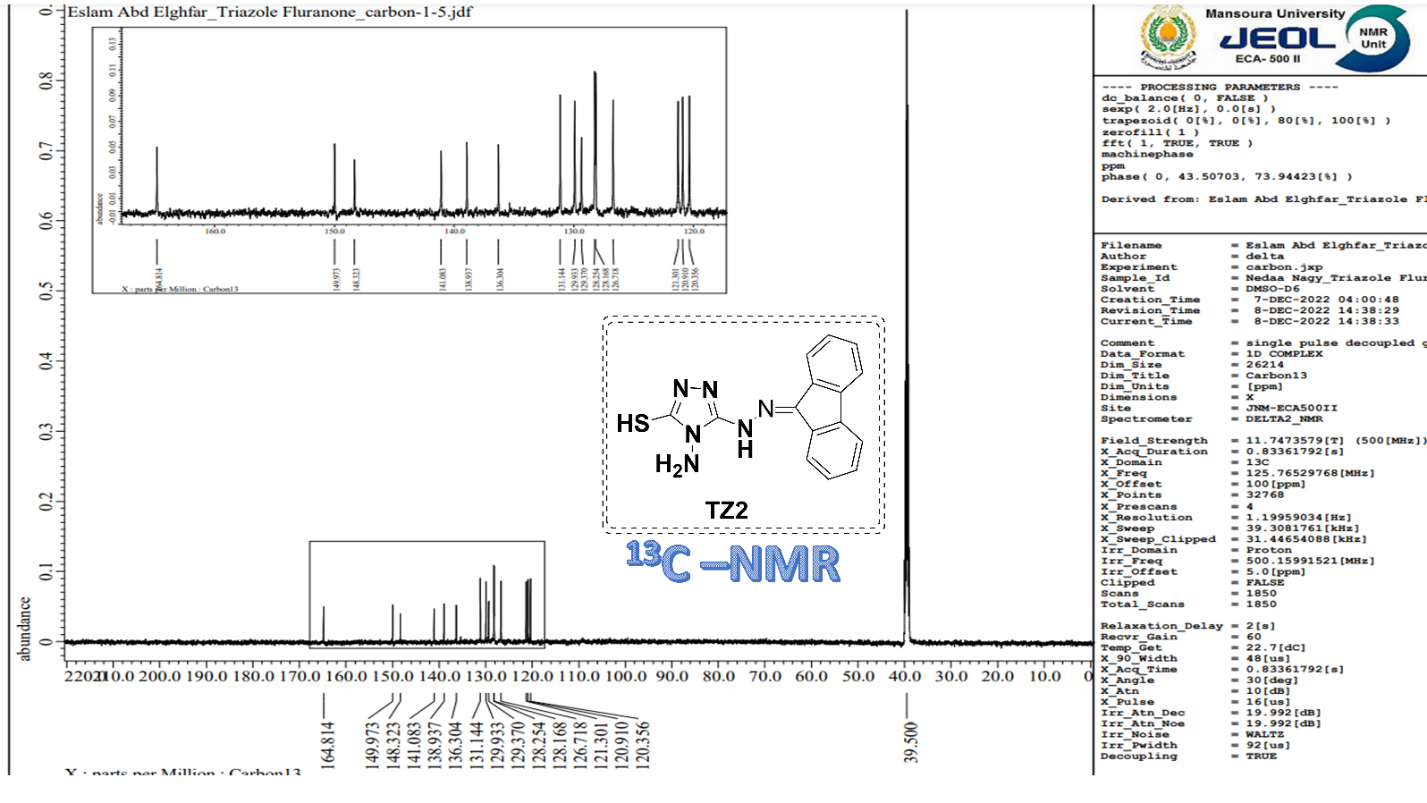
**S6**. ^13^C-NMR spectrum of compound **TZ2**

|  |  |
| --- | --- |
|  |  |
|  |  |

**S7.** Different adsorption isotherms of **TZ1** and **TZ2** for the corrosion of CS in 1 M HCl at 25℃.

**Table S1**. Regression coefficient, slope, and intercept for different adsorption isotherm of **TZ1** and **TZ2**.

| Adsorption isotherms | Inhibitor | Adsorption parameters | | |
| --- | --- | --- | --- | --- |
|  |  | Regression coefficient (R^2^) | Slope | Intercept |
| **Langmuir** | **TZ1** | 0.99684 | 1.11435 | 4.92187E-6 |
|  | **TZ2** | 0.99424 | 1.30196 | 8.96762E-6 |
| **Freundlich** | **TZ1** | 0.93783 | 0.08811 | 0.28785 |
|  | **TZ2** | 0.94324 | 0.13309 | 0.3912 |
| **Temkin** | **TZ1** | 0.92382 | 0.15902 | 0.19184 |
|  | **TZ2** | 0.92664 | 1.49625 | 1.48683 |
| **Frumkin** | **TZ1** | 0.96344 | 6.73356 | -8.46801 |
|  | **TZ2** | 0.97138 | 8.542 | -10.59602 |
| **Florry-Huggins** | **TZ1** | 0.84245 | 2.30383 | 5.9332 |
|  | **TZ2** | 0.8669 | 3.32267 | 5.71703 |
| **El-Awady** | **TZ1** | 0.87807 | 0.42511 | 2.47917 |
|  | **TZ2** | 0.91243 | 0.36253 | 1.85546 |
